# Human neural stem cells alleviate Alzheimer-like pathology in a mouse model

**DOI:** 10.1186/s13024-015-0035-6

**Published:** 2015-08-21

**Authors:** Il-Shin Lee, Kwangsoo Jung, Il-Sun Kim, Haejin Lee, Miri Kim, Seokhwan Yun, Kyujin Hwang, Jeong Eun Shin, Kook In Park

**Affiliations:** Department of Pediatrics, Severance Children’s Hospital, Yonsei University College of Medicine, 50-1 Yonsei-Ro, Seodaemun-Gu, Seoul 120-752 South Korea; Brain Korea 21 Plus Project for Medical Science, Yonsei University College of Medicine, 50-1 Yonsei-Ro, Seodaemun-Gu, Seoul 120-752 South Korea

**Keywords:** Alzheimer’s disease, Human neural stem cells, Transplantation, Trophic factors, Glycogen synthase kinase 3β (GSK3β), Anti-inflammation

## Abstract

**Background:**

Alzheimer’s disease (AD) is an inexorable neurodegenerative disease that commonly occurs in the elderly. The cognitive impairment caused by AD is associated with abnormal accumulation of amyloid-β (Aβ) and hyperphosphorylated tau, which are accompanied by inflammation. Neural stem cells (NSCs) are self-renewing, multipotential cells that differentiate into distinct neural cells. When transplanted into a diseased brain, NSCs repair and replace injured tissues after migration toward and engraftment within lesions. We investigated the therapeutic effects in an AD mouse model of human NSCs (hNSCs) that derived from an aborted human fetal telencephalon at 13 weeks of gestation. Cells were transplanted into the cerebral lateral ventricles of neuron-specific enolase promoter-controlled APPsw-expressing (NSE/APPsw) transgenic mice at 13 months of age.

**Results:**

Implanted cells extensively migrated and engrafted, and some differentiated into neuronal and glial cells, although most hNSCs remained immature. The hNSC transplantation improved spatial memory in these mice, which also showed decreased tau phosphorylation and Aβ42 levels and attenuated microgliosis and astrogliosis. The hNSC transplantation reduced tau phosphorylation via Trk-dependent Akt/GSK3β signaling, down-regulated Aβ production through an Akt/GSK3β signaling-mediated decrease in BACE1, and decreased expression of inflammatory mediators through deactivation of microglia that was mediated by cell-to-cell contact, secretion of anti-inflammatory factors generated from hNSCs, or both. The hNSC transplantation also facilitated synaptic plasticity and anti-apoptotic function via trophic supplies. Furthermore, the safety and feasibility of hNSC transplantation are supported.

**Conclusions:**

These findings demonstrate the hNSC transplantation modulates diverse AD pathologies and rescue impaired memory via multiple mechanisms in an AD model. Thus, our data provide tangible preclinical evidence that human NSC transplantation could be a safe and versatile approach for treating AD patients.

**Electronic supplementary material:**

The online version of this article (doi:10.1186/s13024-015-0035-6) contains supplementary material, which is available to authorized users.

## Background

Alzheimer’s disease (AD), which is characterized by memory loss, progressive cognitive decline, and neuropsychiatric impairment [1], is a devastating neurological disorder that occurs predominantly after 65 years of age. The pathology observed in the AD brain includes aberrant accumulation of amyloid-β (Aβ) and hyperphosphorylated tau, which disturb synaptic function, neuronal homeostasis, and axonal stability, resulting in neuronal loss [1, 2]. Moreover, chronically activated microglia and reactive astrocytes in the brain stimulate deleterious effects through the release of pro-inflammatory cytokines and nitric oxide/reactive oxygen species [2, 3]. To date, the diverse drugs used clinically to treat AD provide temporary symptomatic relief, but do not fundamentally alter the progress of the disease [4]. Therefore, although Aβ-targeted therapies, tau-associated therapies, and anti-inflammatory drugs have been developed to focus on the molecular pathogenesis underlying AD [5–7], an effective treatment has not been established.

Neural stem cells (NSCs) from distinct spatiotemporal neural tissues of mammalian species have the capacity for self-renewal and can give rise to neurons, astrocytes, and oligodendrocytes [8–10]. Human NSCs (hNSCs) isolated from fetal CNS tissue, typically at approximately 6–22 weeks of gestation, are grown as neurospheres in the presence of epidermal growth factor, fibroblast growth factor-2 (FGF-2), or both [11–14]. The hNSCs transplanted into a diseased or injured CNS not only robustly migrate toward and engraft within areas of discrete abnormalities, but also replace dysfunctional tissue and promote survival of injured cells and endogenous tissue repair [15–20]. Therefore, hNSC-based therapy may provide a substantial benefit by preventing, obstructing, or reversing AD. Recently, several studies showed that NSC grafting into the hippocampus improved the cognitive deficits by the recovery of synaptic plasticity and attenuating the expression of proinflammatory cytokines in AD models [21–25]. However, it is still not clear how NSC transplantation regulates molecular signaling pathways related to AD-like neuropathologies.

In this study, we explored the therapeutic potential of human fetal brain-derived NSCs in NSE/APPsw transgenic mice harboring APP Swedish mutant under the control of the rat neuron-specific enolase (NSE) promoter. The transgenic mice exhibit increased intracellular Aβ deposition and tau phosphorylation, and develop spatial memory deficits at 12 months of age [26]. Herein, we determined whether hNSC transplantation improved behavioral deficits in NSE/APPsw transgenic mice, and characterized the distribution, engraftment, and the differentiation patterns of cells implanted in mouse recipients. We further investigated whether hNSC transplantation could modulate Aβ and tau pathology, neuroinflammation, and synaptic plasticity.

## Results

### Engraftment, migration, and differentiation of human NSCs following transplantation

**Fig. 1:**
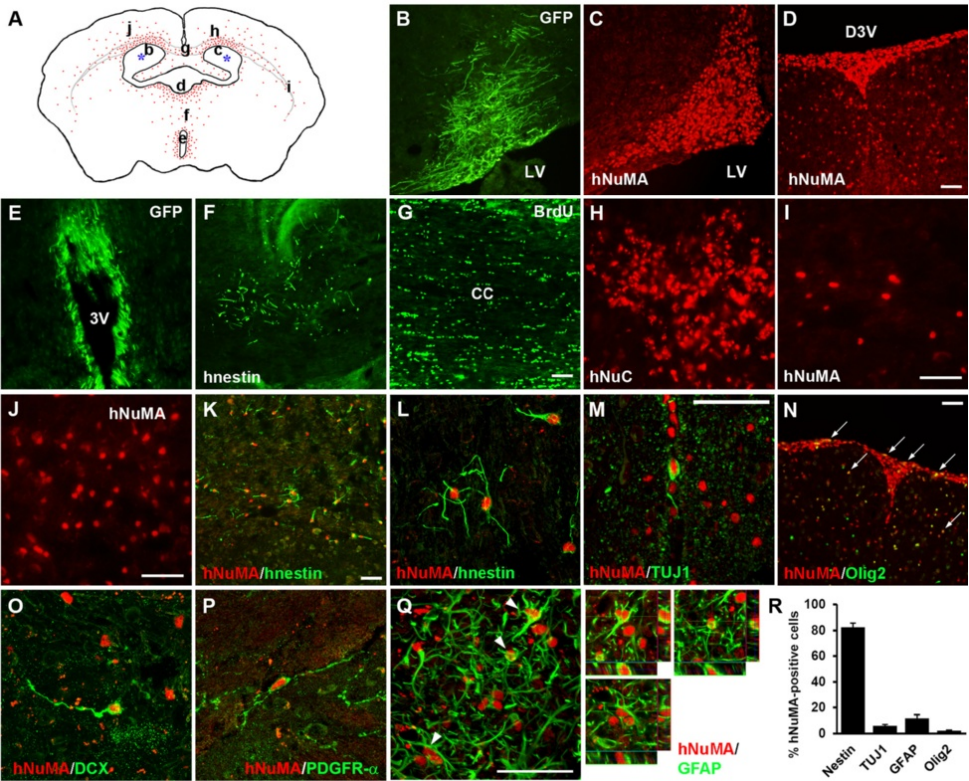
Transplantation of hNSCs into NSE/APPsw transgenic mice. **a** Schematic drawing of a representative coronal brain section, illustrating the wide distribution of hNuMA^+^ grafted cells (red dots). Asterisks indicate the transplantation sites. Lowercase letters represent the site captured in the respective photomicrographs shown in (**b–j**). **b**–**e** GFP-expressing hNSCs (**b** and **e**) or hNuMA^+^ cells (**c** and **d**) are largely engrafted from the LV or third ventricle (3 V) to the SVZ, and from dorsal 3 V (D3V) to the thalamus and hypothalamus. **f** Some hnestin^+^ cells are detected in the thalamus. **g**–**j** The grafted cells migrate along the white matter tracts: BrdU^+^ cells in the corpus callosum (cc; **g**); hNuC^+^ cells in the cingulum (**h**); and hNuMA^+^ cells in the external capsule (**i**), and toward the cortex (**j**). **k**–**n** Most hNuMA^+^ cells express hnestin in the cortex (**k**) and external capsules (**l**), whereas a few hNuMA^+^ cells are co-localized with either TUJ1^+^ or Olig2^+^ cells in the SVZ (**m**) and thalamus (**n**), respectively. Arrows in N indicate hNuMA/Olig2 double-labeled cells. **o**–**q** A few hNuMA^+^ cells express DCX (**o**) or PDGFR-α (**p**) in the white matter tracts, and GFAP (**q**) in the cortex. Arrowheads in Q indicate co-localization of hNuMA and GFAP, representing z-stack confocal images (three small images in the right panel of **q**). **r** Differentiation patterns of grafted hNSCs in transgenic mice (*n* = 4, where n is the number of mice). In **k**–**m**, **o**, and **p**, z-stack images are built and compiled to maximal intensity projections. Scale bars, 50 μm

Non-engineered hNSCs, lenti-GFP-transduced hNSCs (Additional file 1: Figure S1), and 2 μM 5-bromo-2′-deoxyuridine (BrdU)-labeled hNSCs were prepared to identify grafted cells in multiple brain locations of NSE/APPsw transgenic mice at 7 weeks post-transplantation. The grafted human nuclear matrix (hNuMA)^+^, human nuclear antigen (hNuC)^+^, human nestin (hnestin)^+^, GFP^+^, or BrdU^+^ cells were widely distributed from the transplantation site to the subventricular zone (SVZ), white matter tracts, striatum, thalamus, hypothalamus, and cortex, although a large proportion of the grafted cells was localized to the SVZ (Fig. 1a-j). The majority of hNuMA^+^ cells expressed hnestin, an immature cell marker (Fig. 1k and l), whereas others were co-localized with the early neuronal cell marker neuronal class III β-tubulin (TUJ1) in the SVZ (Fig. 1m), the oligodendroglial progenitor cell marker Olig2 in the SVZ and thalamus (Fig. 1n), the neuroblast marker doublecortin (DCX) in the external capsule (Fig. 1o), the oligodendroglial progenitor cell marker platelet-derived growth factor receptor-α (PDGFR-α) in the external capsule (Fig. 1p), and the astrocyte marker glial fibrillary acidic protein (GFAP) in the cortex (Fig. 1q). Quantification revealed that most grafted cells expressed hnestin (82.4 ± 3.3 %), whereas some cells differentiated into TUJ1^+^ neurons (5.8 ± 0.9 %), Olig2^+^ oligodendrocyte progenitors (2.3 ± 0.4 %), and GFAP^+^ astrocytes (11.7 ± 2.8 %) (Fig. 1r). These data demonstrated that human fetal brain-derived NSCs showed extensive migration, robust engraftment, and differentiation into three CNS neural cell types, albeit that most cells remained in an immature state following transplantation. Additionally, some donor-derived cells still survived in the SVZ, third ventricle, and thalamus adjacent to the site of transplantation, and differentiated into neuronal or glial cells, albeit that most remained immature even when analyzed at 3 months following implantation. (Additional file 2: Figure S2). Thus, implanted hNSCs into the AD model migrate and survive for a prolonged period, although the number of grafted cells reduced gradually over time.

### Human NSC transplantation improves spatial memory

**Fig. 2:**
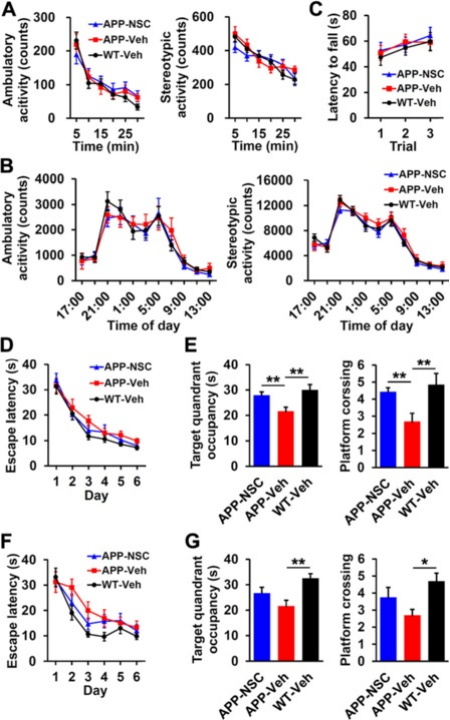
Behavioral phenotypes of hNSC- or vehicle-injected NSE/APPsw transgenic mice and vehicle-injected wild-type mice. **a** Ambulatory and stereotypic activity of the three groups in a 30 min open field test (APP-NSC, *n* = 24; APP-Veh, *n* = 20; and WT-Veh, *n* = 24). **b** Ambulatory and stereotypic locomotor activity over 22 h (APP-NSC, *n* = 12; APP-Veh, *n* = 8; and WT-Veh, *n* = 14). **c** Latency to fall in the accelerating rotarod task (APP-NSC, *n* = 26; APP-Veh, *n* = 20; and WT-Veh, *n* = 23). **d** and **e** Results from the water maze test 6 weeks after hNSC or vehicle injection. The latency to reach the hidden platform (escape latency) during the learning phase was recorded **d**. Target quadrant occupancy and platform crossing during the probe trial were measured in the three groups (APP-NSC, *n* = 21; APP-Veh, *n* = 19; and WT-Veh, *n* = 19; **e**). **f** and **g** Results from the water maze test 12 weeks post-transplantation. Escape latency during the learning phase in the three groups (**f**). Target quadrant occupancy and platform crossing during the probe trial (APP-NSC, *n* = 12; APP-Veh, *n* = 10; and WT-Veh, *n* = 10; **g**). The number of mice (n) in each group is indicated. All data represent mean ± SEM. Error bars indicate ± SEM. *p < 0.05, **p < 0.01

Mice were grouped into hNSC- (APP-NSC) or vehicle (Hank’s balanced salt solution–10 mM HEPES [H-H] buffer only)-injected (APP-Veh) NSE/APPsw transgenic mice and vehicle-injected wild-type mice (WT-Veh). The open field test and the accelerating rotarod task were performed at 5 weeks post-transplantation. No changes in ambulatory or stereotypic activity were observed in the open field test, which is affected by exploratory activity and emotionality (Fig. 2a), and interactions of day or night locomotor activity (Fig. 2b), respectively, among the groups. In the accelerating rotarod task, which reflects motor coordination, the three groups showed no differences in latency to fall (Fig. 2c). In the Morris water maze test at 6 weeks post-transplantation, the groups revealed no differences during the learning phase (Fig. 2d). However, target quadrant occupancy was significantly lower, and fewer platform crossings were observed in the APP-Veh group (21.63 ± 1.58 s and 2.68 ± 0.49, respectively) than in the WT-Veh group (30.04 ± 2.11 s and 4.84 ± 0.67, respectively; *p* < 0.01; Fig. 2e). Moreover, the APP-NSC group showed a significant increase in these parameters (27.88 ± 1.36 s and 4.43 ± 0.25, respectively) compared with the APP-Veh group (*p* < 0.01; Fig. 2e). Additionally, in the water maze test at 12 weeks post-transplantation, the overall learning phase was not different among the three groups (Fig. 2f). The APP-Veh group showed a significant decrease in target quadrant occupancy and platform crossing compared with the WT-Veh group (21.58 ± 2.70 versus 32.58 ± 1.76 s, *p* < 0.01, and 2.70 ± 0.34 versus 4.70 ± 0.48, *p* < 0.05, respectively; Fig. 2g). However, hNSC grafting tended to increase these values in transgenic mice, although this increase was not statistically significant (26.70 ± 2.31 s and 3.75 ± 0.58, respectively; Fig. 2g). These results indicated that hNSC transplantation ameliorated the impaired spatial memory without causing the mice to exhibit atypical locomotion, spontaneous motor alterations, or aberrant motor coordination but did not prevent long-term progressive cognitive deficits in transgenic mice.

### Human NSC transplantation inhibits tau phosphorylation

**Fig. 3:**
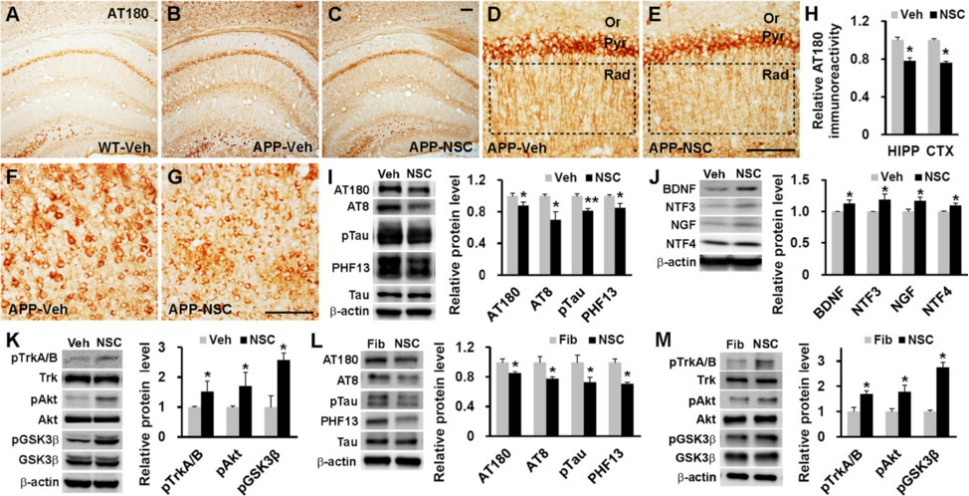
hNSC transplantation reduces tau phosphorylation via Trk-induced Akt/GSK3β signaling in the NSE/APPsw transgenic mouse brain. **a**–**c** Photomicrographs of AT180 immunostaining in the brains of vehicle-injected wild-type (WT-Veh; **a**), vehicle-injected transgenic (APP-Veh; **b**), and hNSC-injected transgenic (APP-NSC; **c**) mice. **d**–**g** AT180 immunoreactivity in the stratum radiatum of the hippocampal CA1 region (HIPP; inside the dotted rectangle in **d** and **e**) and the posterior parietal cortex (CTX; **f** and **g**) in hNSC- and vehicle-injected transgenic mice. Or, oriens layer; Pyr, pyramidal cell layer; Rad, stratum radiatum. **h** Immunohistochemical image analyses of AT180 immunoreactivity comparing hNSC graft (NSC, *n* = 4) and vehicle injection (Veh, *n* = 4) in the transgenic mice. **i** Levels of phosphorylated tau (AT180, AT8, pTau, and PHF13) on western blot image analyses in brains of vehicle-injected (*n* = 5) and hNSC-injected (*n* = 6) transgenic mice. **j** Levels of neurotrophins (BDNF, NTF3, NGF, and NTF4) on western blot image analyses in brains of vehicle-injected (*n* = 3) and hNSC-injected (*n* = 4) transgenic mice. **k** Levels of phosphorylated TrkA/B, Akt and GSK3β using western blot analysis on brains of vehicle-injected (*n* = 3) and hNSC-injected (*n* = 3) transgenic mice. **l** and **m**Relative levels of phosphorylated tau (**l**) and kinases related to Trk-dependent Akt/GSK3β signaling (**m**) using western blot image analyses from Aβ42-treated PC12 cells in the presence of hNSC-derived CM (NSC, *n* = 3) and fibroblast-derived CM (Fib, *n* = 3). Scale bars, 100 μm. The number of mice (n) in H-K is indicated. The number of experiments (n) in L and M is indicated. All data represent mean ± SEM. Error bars indicate ± SEM. *p < 0.05, **p < 0.01

We measured the level of phosphorylated tau (AT180) in hNSC- and vehicle-injected NSE/APPsw transgenic mice because aberrant phosphorylation of tau worsens cognitive function via axonal and synaptic disruption in AD [2, 6]. Vehicle-injected transgenic mice showed stronger AT180 immunoreactivity than vehicle-injected wild-type mice (Fig. 3a and b), consistent with pervious report [26]. Notably, hNSC-transplanted transgenic mice showed a significant decrease in the intensity of AT180 immunoreactivity in the hippocampal CA1 and cortical regions compared with their vehicle-injected cohorts (*p* < 0.05; Fig. 3b-h). In addition, western blot indicated that levels of tau phosphorylation were significantly attenuated by hNSC transplantation in transgenic mouse brain (AT180 [Thr231], *p* < 0.05; AT8 [Ser202], *p* < 0.05; pTau [Ser404], *p* < 0.01; PHF13 [Ser396], *p* < 0.05; Fig. 3i). Therefore, hNSC transplantation reduced tau phosphorylation in transgenic mouse brain, suggesting that grafted hNSCs widely modulated tau phosphorylation in a diffusible fashion because the hNSCs were rarely found in the hippocampus.

### Human NSC transplantation activates Trk-dependent Akt/GSK3β signaling

In this study, hNSCs expressed trophic factors including neurotrophins (BDNF, NTF3, NTF4, NGF, VEGF, FGF2, and GDNF) that induce Trk-dependent Akt activation [27] and secreted them into the culture media (Additional file 3: Figure S3). Moreover, hNSC transplantation significantly increased the levels of neurotrophins in the brains of transgenic mice compared with those in their vehicle-injected cohorts (*p* < 0.05; Fig. 3j). Furthermore, hNSC transplantation induced significantly higher phosphorylation levels of TrkA/B and Akt, and markedly elevated the level of GSK3β phosphorylation (*p* < 0.05, respectively; Fig. 3k), suggesting that tau phosphorylation was modulated by augmented neurotrophin-mediated Trk-dependent Akt/GSK3β signaling in transgenic mice, because GSK3β is the major kinase phosphorylating tau and its activity is suppressed by Akt-mediated phosphorylation at Ser9 [28, 29]. Next, hNSC- or human foreskin fibroblast-derived conditioned media (CM)-treated PC12 cells were incubated with soluble Aβ42 because abundant Aβ promotes tau phosphorylation in the cells [30]. The presence of hNSC-derived CM in Aβ42-treated cells significantly hampered tau phosphorylation and markedly induced phosphorylation of TrkA/B, Akt, and GSK3β compared with those in the presence of fibroblast-derived CM (*p* < 0.05, respectively; Fig. 3l and m). Therefore, these results suggested that hNSCs modulated GSK3β activity through Trk-induced Akt activation to ultimately decrease tau phosphorylation in transgenic mice.

### Human NSC transplantation leads to reduced Aβ42 levels

**Fig. 4:**
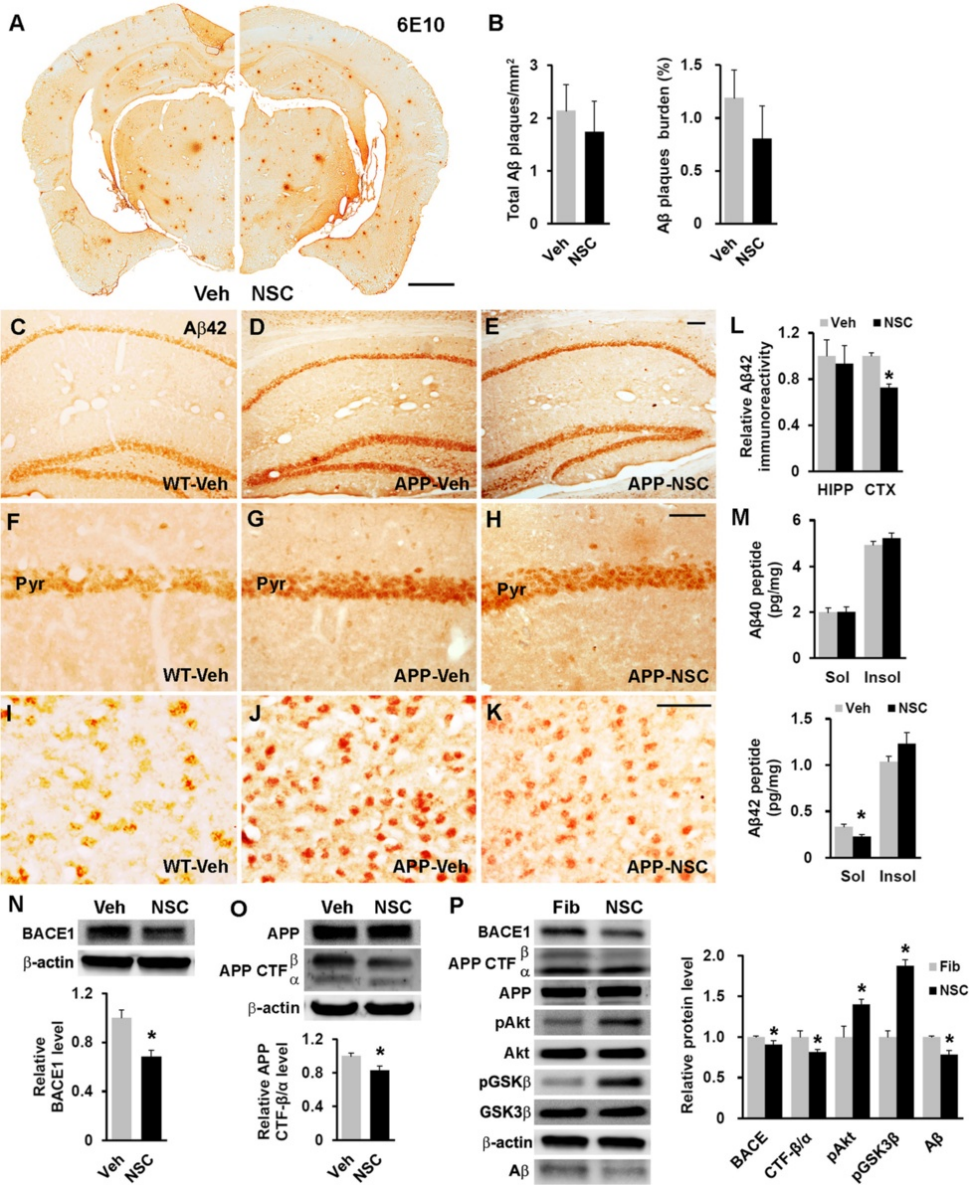
hNSC transplantation decreases brain intracellular soluble Aβ42 levels by regulating BACE1 in NSE/APPsw transgenic mice. **a** Photomicrographs of Aβ plaque (6E10) staining in hNSC-injected (NSC) and vehicle-injected (Veh) transgenic mice. Scale bar, 1 mm. **b** The number of Aβ plaques per unit area (left) and the percent area of the Aβ plaque load (right) between hNSC graft (NSC) and vehicle injection (Veh; *n* = 3 per group). **c**–**l** Representative images of Aβ42 immunostaining in the brains of vehicle-injected wild-type mice (WT-Veh; **c**), and of vehicle-injected (APP-Veh; **d**) and hNSC-injected (APP-NSC; **e**) transgenic mice. Aβ42 immunostaining of each group in the pyramidal cell layer of the hippocampal CA1 region (HIPP; **f**–**h**) and the posterior parietal cortex (CTX; **i**–**k**). The relative levels of Aβ42 immunostaining in vehicle-injected (Veh) and hNSC-injected (NSC) transgenic mice (*n* = 4 per group; **l**). Scale bar, 100 μm (**e** and **h**) and 50 μm (**k**). **m** Levels of detergent-soluble (Sol) Aβ40/42 and detergent-insoluble (Insol) Aβ40/42 in the brains of hNSC-injected (NSC) and vehicle-injected (Veh) transgenic mice using ELISA kits (*n* = 4 per group). **n** and **o** Relative levels of BACE1 (NSC, *n* = 6; Veh, *n* = 5; **n**) and APP CTF-β/CTF-α (NSC, *n* = 4; Veh, *n* = 3; **o**) using western blot analyses in brains of hNSC-injected (NSC) and vehicle-injected (Veh) transgenic mice. **p** APPsw-expressing SK-N-MC cells treated with hNSC-derived (NSC) and fibroblast-derived (Fib) CM (*n* = 3 per group, where n is the number of experiments). Western blot image analysis of Aβ in the culture media of these cells, and BACE1, APP CTF-β/CTF-α, and phosphorylated Akt and GSK3β in cells of both groups. The number of mice (n) in (**b)** and (**l**-**o)** is indicated. All data represent mean ± SEM. Error bars indicate ± SEM. *p < 0.05

We compared the area and amount of the Aβ load in hNSC- and vehicle-injected NSE/APPsw transgenic mice because Aβ is directly associated with cognitive decline in AD [1, 2]. Aβ plaque staining showed no statistically significant differences in the area or number of Aβ plaques, although the area of Aβ plaques was nonsignificantly lower in cell-injected mice (total Aβ plaques/mm^2^, 1.74 ± 0.58 versus 2.14 ± 0.50; Aβ plaque burden, 0.80 ± 0.32 versus 1.19 ± 0.26; Fig. 4a and b). Additionally, we examined intracellular Aβ42 immunoreactivity, because a marked accumulation of intracellular Aβ in some AD models elicits synaptic dysfunction and cognitive deficits before or without the aggressive development of Aβ plaques [31–33]. NSE/APPsw transgenic mice exhibit more intensive intracellular Aβ42 immunoreactivity compared with wild-type mice (Fig. 4c, d, f, g, i, and j). The hNSC transplantation significantly decreased Aβ42 immunoreactivity in the cortex (*p* < 0.05) but not in the hippocampus compared with that in the vehicle-injected cohorts (Fig. 4d, e, g, h, and j–l). Moreover, we found that detergent-soluble Aβ42 was significantly decreased in the hNSC-transplanted mice compared with that in the vehicle-injected mice (0.23 ± 0.02 versus 0.34 ± 0.03 pg/mg, *p* < 0.05), whereas detergent-soluble Aβ40 and insoluble Aβ40/42 were indistinguishable in both groups as assessed in whole brain extracts of transgenic mice (soluble Aβ40, 2.01 ± 0.21 versus 1.98 ± 0.19 pg/mg; insoluble Aβ40, 5.23 ± 0.22 versus 4.93 ± 0.16 pg/mg; and insoluble Aβ42, 1.23 ± 0.12 versus 1.04 ± 0.06 pg/mg; Fig. 4m). Therefore, hNSC transplantation appeared to play a key role in the reduced level of intracellular soluble Aβ42, albeit only slightly, in transgenic mouse brain.

### Human NSCs alter APP processing by modulating BACE1 expression

We investigated whether hNSC transplantation affected the expression of BACE1, the essential β-secretase for APP processing, leading to Aβ production. The brain levels of BACE1 were substantially reduced in hNSC-injected NSE/APPsw transgenic mice compared with those in their vehicle-injected cohorts (*p* < 0.05; Fig. 4n), consistent with reports indicating that Akt activation or GSK3β inactivation reduce BACE1 levels [34, 35]. Moreover, hNSC transplantation induced a significant decrease in the level of APP C-terminal fragment β (CTF-β) relative to APP C-terminal fragment α (CTF-α) compared with that following vehicle injections in transgenic mouse brains (*p* < 0.05; Fig. 4o), reflecting the decrease in Aβ production. Furthermore, the expression of membrane metallo endopeptidase (*Mme*) and insulin degrading enzyme (*Ide*), known as main Aβ degrading enzymes, showed no significant difference between hNSC- and vehicle-injected transgenic mice (Additional file 4: Figure S4A). Additionally, although hNSCs expressed various Aβ-degrading enzymes *in vitro*, there were no differences in the levels of Aβ42 in the media containing 1 μM soluble Aβ42 peptides between wells with and without incubation with hNSCs. (Additional file 4: Figure S4B). Next, APPsw-expressing SK-N-MC human neuroblastoma cells were incubated with the hNSC- or fibroblast-derived CM. Notably, hNSC-derived CM-treated cells showed substantially lower levels of BACE1 and APP CTF-β/CTF-α than cells treated with fibroblast-derived CM (*p* < 0.05), leading to a decrease of Aβ in the culture media (*p* < 0.05) and higher phosphorylation of Akt and GSK3β (*p* < 0.05) (Fig. 4p). Taken together, these results suggested that grafted hNSCs were involved in APP processing through Akt/GSK3β signaling-mediated BACE1 modulation in transgenic mouse brain.

### Human NSC transplantation decreases astrogliosis and microgliosis

**Fig. 5:**
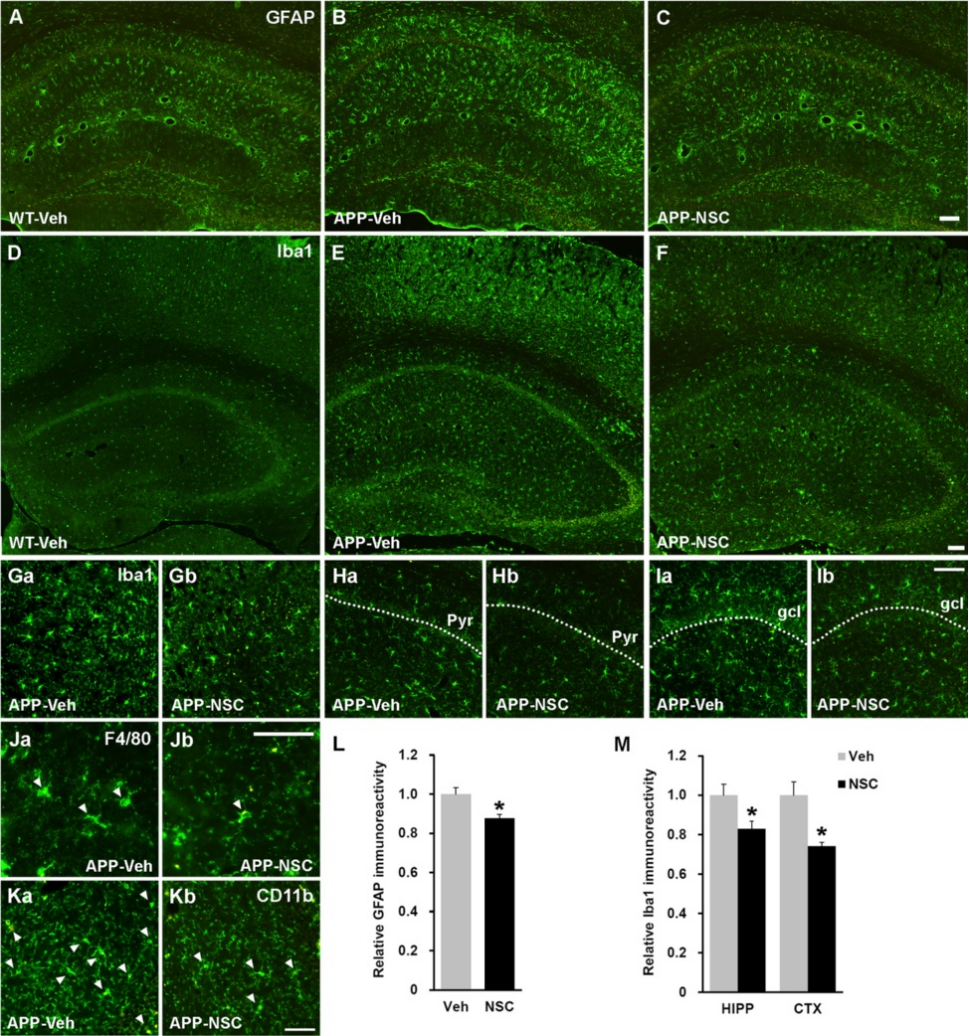
hNSC transplantation suppresses astrogliosis and microgliosis in the NSE/APPsw transgenic mouse brain. **a**–**c** Representative images of GFAP^+^ astrogliosis in the hippocampus of vehicle-injected wild-type mice (WT-Veh; **a**), and of vehicle-injected (APP-Veh; **b**) and hNSC-injected (APP-NSC; **c**) transgenic mice, at 7 weeks post-transplantation. Scale bar, 100 μm (**c**). **d**–**f** Representative images of Iba1^+^ microgliosis in the brains of vehicle-injected wild-type mice (**d**), and of vehicle-injected (**e**) and hNSC-injected (**f**) transgenic mice, at 7 weeks post-transplantation. Scale bar, 100 μm (**f**). **Ga**–**Ib** The hNSC transplantation substantially decreases the numbers of Iba1^+^ cells in the cortex (**Gb**) and hippocampus (**Hb** and **Ib**) of transgenic mice compared with those in their vehicle-injected cohorts (**Ga**, **Ha**, and **Ia**). Scale bar, 100 μm (**Ib**). gcl, granular cell layer in the dentate gyrus of the hippocampus. **Ja**–**Kb** The numbers of F4/80^+^ (**Ja** and **Jb**) and CD11b^+^ microglia (**Ka** and **Kb**) appear to decrease in the hippocampus of hNSC-injected transgenic mice compared with those in their vehicle-injected cohorts. Scale bar, 50 μm (**Jb** and **Kb**). **l** Relative levels of the optical density of GFAP immunoreactivity in the hippocampus of hNSC-injected (NSC, *n* = 3) and vehicle-injected (Veh, *n* = 3) transgenic mice. **m** Relative levels of Iba1 immunoreactivity, quantified as a percentage of the area occupied using ImageJ, in the cortex (CTX) and hippocampus (HIPP) of hNSC- and vehicle-injected transgenic mice (*n* = 3 per group). The number of mice (n) in L and M is indicated. All data represent mean ± SEM. Error bars indicate ± SEM. *p < 0.05

**Fig. 6:**
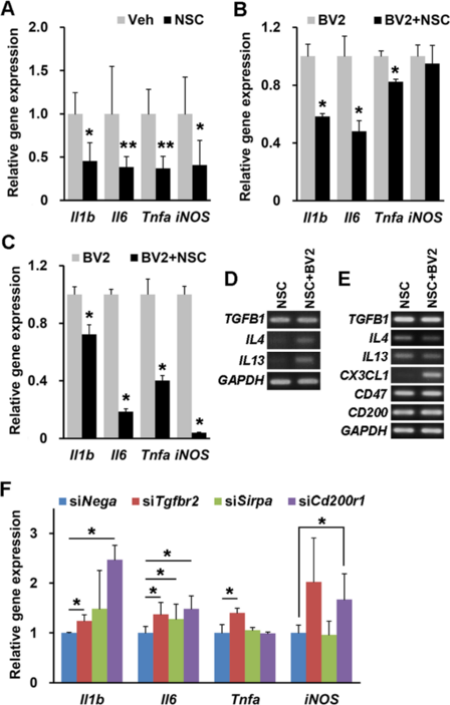
hNSCs reduce the expression of pro-inflammatory mediators. **a** Comparison of the expression of *Il1b*, *Il6*, *Tnfa*, and *iNOS* mRNA levels in the brains of hNSC-injected (NSC, *n* = 7) and vehicle-injected (Veh, *n* = 6) NSE/APPsw transgenic mice. **b** and **d** The expression of *Il1b*, *Il6*, and *Tnfa* in LPS-stimulated BV2 microglial cells co-cultured with hNSCs (BV2 + NSC) compared with that in single cultures of LPS-stimulated BV2 cells (BV2) on Transwell permeable supports (*n* = 3 per group; **b**). The hNSCs co-cultured with BV2 cells (NSC + BV2) express *TGFB1*, *IL4*, and *IL13* (**d**). **c** and **e** In mixed cultures, the change of *Il1b*, *Il6*, *Tnfa*, and *iNOS* expression in LPS-stimulated BV2 cells (BV2 + NSC) co-cultured with hNSCs compared with that in single cultures of LPS-stimulated BV2 cells (BV2; *n* = 3 per group; **c**). The hNSCs co-cultured with BV2 cells (NSC + BV2) express *TGFB1*, *IL4*, *IL13*, *CX3CL1*, *CD47*, and *CD200* (**e**). **f** The relative expression of mRNA for *Il1b*, *Il6*, *Tnfa,* and *iNOS* in *Tgfbr2*-, *Sirpa*-, and *Cd200r1*-knockdown BV2 cells co-cultured with hNSCs compared with that in non-functioning negative siRNA (siNega)-transfected BV2 cells in mixed co-culture in the presence of LPS (*n* = 3 per group). The number of mice (n) in A is indicated. The number of experiments (n) in (**b**, **c**), and (**f**) is indicated. All data represent mean ± SEM. Error bars indicate ± SEM. *p < 0.05, **p < 0.01

Because activation of astrocytes and microglia in mutant APP-expressing mice aggravate neurodegenerative milieu by pro-inflammatory mediators, resulting in cognitive impairment [36, 37], we performed staining with GFAP and the microglial markers Iba1, CD11b, and F4/80 to examine the effects of hNSC transplantation on neuroinflammation in NSE/APPsw transgenic mice. Astrogliosis and microgliosis were markedly enhanced in vehicle-injected transgenic mice compared with that in vehicle-injected wild-type mice (Fig. 5a, b, d and e), whereas hNSC transplantation significantly reduced the level of GFAP and Iba1 immunoreactivity in transgenic mice (*p* < 0.05; Fig. 5b, c, e-i, l and m), respectively. Additionally, the numbers of CD11b^+^ and F4/80^+^ cells were decreased in hNSC-injected transgenic mice compared with those in their vehicle-injected cohorts (Fig. 5j and k). Furthermore, hNSC transplantation significantly down-regulated the expression levels of pro-inflammatory mediators (*Il1b*, *Il6*, *Tnfa*, and *iNOS*) in transgenic mice (*p*_*Il1b*_ < 0.05, *p*_*Il6*_ < 0.01, *p*_*Tnfa*_ < 0.01, and *p*_*iNOS*_ < 0.05; Fig. 6a). Collectively, these data indicated that hNSC transplantation had an anti-inflammatory effect against astrogliosis and microgliosis in transgenic mice.

### Human NSCs attenuate microglial activation through cell-to-cell contact and secretory molecules

To elucidate the immunomodulatory effects of hNSCs on microglia, we indirectly or directly cultured lipopolysaccharide (LPS)-activated BV2 microglial cells with hNSCs. Firstly, BV2 cells co-cultured in Transwell showed a significant reduction in the levels of *Il1b*, *Il6*, and *Tnfa*, but not *iNOS*, compared with BV2 cells cultured alone (*p*_*Il1b*_ < 0.05, *p*_*Il6*_ < 0.05, *p*_*Tnfa*_ < 0.05, and *p*_*iNOS*_ = 0.827; Fig. 6b). Next, BV2 cells separated from mixed-culture in the presence of LPS (Additional file 5: Figure S5A) had significantly lower expression of *Il1b*, *Il6*, *Tnfa*, and *iNOS* than BV2 cells cultured alone (*p* < 0.05; Fig. 6c). Additionally, we found that co-cultured hNSCs expressed secretory or cell-to-cell contact anti-inflammatory factors (*TGFB1*, *IL4*, *IL13*, *CX3CL1*, *CD47*, and *CD200*) in Transwell and mixed-cultures, respectively (Fig. 6d and e). Lastly, we directly cultured gene-knockdown BV2 cells (Additional file 5: Figure S5B) with hNSCs in the presence of LPS to identify which factors strongly expressed in hNSCs abrogate expression of pro-inflammatory mediators in activated BV2 cells. The BV2 cells with the TGF-β receptor 2 (*Tgfbr2*)-knockdown had significantly increased expression of *Il1b*, *Il6*, and *Tnfa* (*p* < 0.05)*,* whereas signal regulatory protein α (*Sirpa* as the CD47 receptor)-knockdown BV2 cells exhibited significantly increased expression of only *Il6* (*p* < 0.05; Fig. 6f). CD200 receptor 1 (*Cd200r1*)-knockdown BV2 cells showed significantly elevated levels of *Il1b*, *Il6*, and *iNOS* (*p* < 0.05; Fig. 6f). These data indicated that hNSCs attenuated the microglial expression of pro-inflammatory mediators, suggesting that TGF-β1, CD47, and CD200 generated in hNSCs had anti-inflammatory effects on activated microglia.

### Human NSC transplantation enhances synaptic density and prevents cell death

**Fig. 7:**
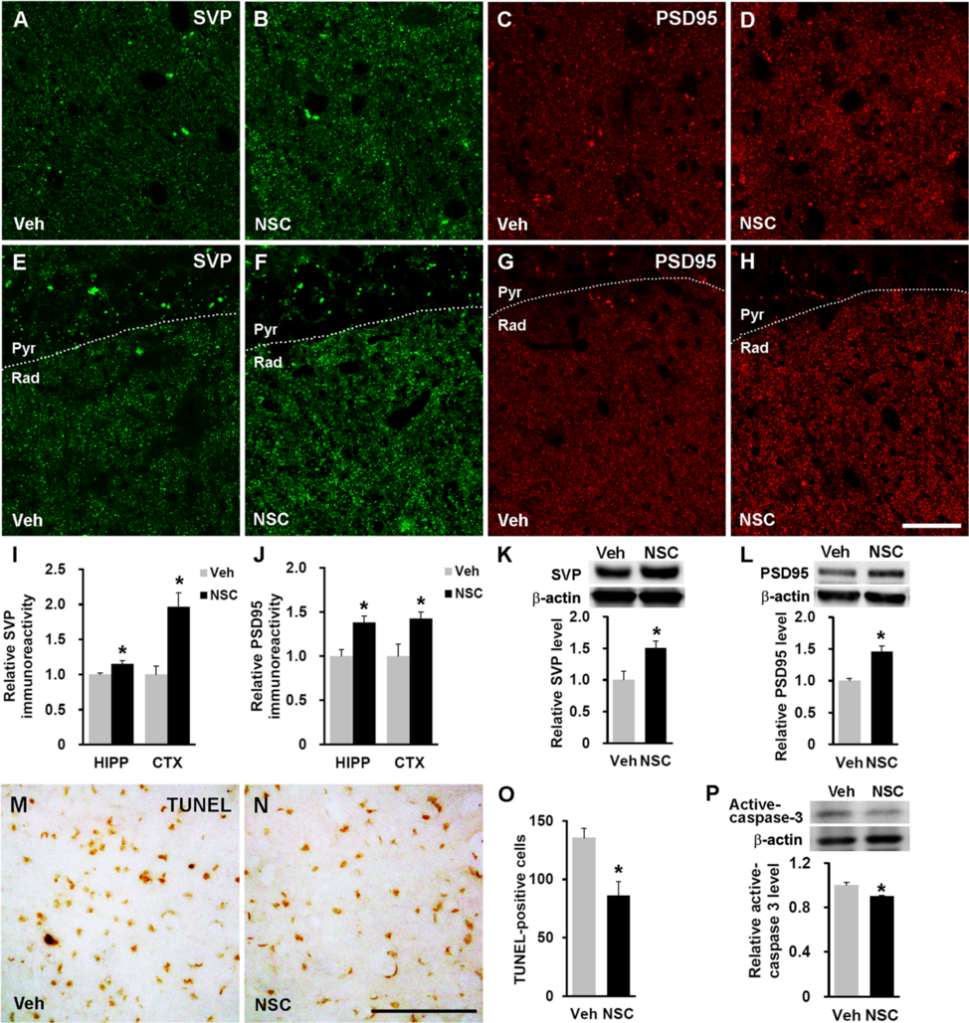
hNSC transplantation increases synaptic density and decreases apoptosis in the NSE/APPsw transgenic mouse brain. **a**-**h** Photomicrographs of SVP and PSD95 immunostaining (green and red puncta, respectively) in the posterior parietal cortex (**a**-**d**) and striatum radiatum of the hippocampal CA1 region (**e**-**f**) in vehicle-injected (Veh; **a**, **c**, **e**, and **g**) and hNSC-injected (NSC; **b**, **d**, **f**, and **h**) transgenic mice at 7 weeks post-transplantation. Scale bar, 25 μm (**h**). **i** and **j** Results of image analyses using confocal microscopy for synaptic density (SVP and PSD95) in the cortex (CTX) and hippocampus (HIPP) comparing hNSC- and vehicle-injected transgenic mice (*n* = 4 per group; I, *n* = 3 per group; **j**). **k** and **l** Western blot analysis of SVP (**k**) and PSD95 (**l**) in both groups (*n* = 3 per group). **m** and **n** Representative images of TUNEL^+^ cells in the posterior parietal cortex of vehicle-injected (**m**) and hNSC- injected (**n**) transgenic mice at 7 weeks post-transplantation. Scale bar, 100 μm (**h**). **o** Quantification of the number of TUNEL^+^ cells of the posterior parietal cortex in both groups (*n* = 3 per group). **p** The relative level of active caspase-3 in the whole brains of hNSC- and vehicle-injected mice on western blot analysis (*n* = 4 per group). The number of mice (n) in (**i**-**l**, **o**, and **p**) is indicated. All data represent mean ± SEM. Error bars indicate ± SEM. *p < 0.05

We analyzed synaptophysin (SVP) and postsynaptic density protein 95 (PSD95) immunoreactivity and levels to examine synaptic changes in hNSC- or vehicle-injected NSE/APPsw transgenic mice. A significant increase of synaptic density (*p* < 0.05 in both SVP and PSD95) and levels (*p* < 0.05 in both SVP and PSD95) were found between hNSC- and vehicle-injected mouse brains (Fig. 7a–l), suggesting that hNSC transplantation attenuated the synaptotoxic properties of Aβ and promoted synaptic plasticity.

We performed a terminal deoxynucleotidyl transferase dUTP nick-end labeling (TUNEL) assay and estimated expression of active caspase-3 to investigate whether hNSC transplantation prevents cell death in transgenic mice. A substantial decrease in the number of TUNEL^+^ cells (85.9 ± 12.4 versus 135.7 ± 8.0, *p* < 0.05) and the level of active caspase-3 (*p* < 0.05) were found in the brains of hNSC-injected mice compared with that in their vehicle-injected cohorts (Fig. 7m–p), demonstrating that hNSC transplantation protected host brain cells against the cytotoxic environment of transgenic mouse.

## Discussion

Because AD affects multiple neural systems and broad regions of the brain, we performed intracerebroventricular transplantation of hNSCs in 13-month-old NSE/APPsw transgenic mice to perpetuate widespread donor cell engraftment, minimize transplantation-associated tissue damage, and leverage endogenous mediators that could elicit migration of implanted cells toward various lesions. We observed that transplanted cells extensively migrated and engrafted into various brain areas except the hippocampus and remained mostly immature although these cells were reliably multipotent, as previously described [12, 38]. Many studies reported that implanted hNSCs predominantly differentiated into astrocytes in various neurological disease models. However, several other studies also observed differentiation failure of engrafted hNSCs following transplantation [15, 20, 22, 39, 40]. The difference in differentiation patterns may be caused by the source of the human cells, culturing techniques, and cell preparation, as well as potential differences between injury models. In fact, engrafted hNSCs into disease models may respond to both cell-intrinsic programming and local environmental factors provided by multiple cellular and acellular components. Thus, better understanding of a broad spectrum of signals that occur in the AD-specific pathologic conditions will be required to gain more insights into the differentiation pattern of grafted cells. These findings suggest that the beneficial effects of hNSC grafting in this AD mouse model are not via cell replacement; consequently, neuronal replacement by grafted cells is unlikely.

In this study, the safety and feasibility of hNSC transplantation were supported. There was no adverse finding over 2–3 months after hNSC implantation into the brain of NSE/APPsw transgenic mice. The transplantation procedure did not result in any harmful side effects; infection, hemorrhage, tumor, atypical locomotion, spontaneous motor alterations, aberrant motor coordination, or any behavioral abnormalities. Regarding mortality following transplantation, all mice groups in this study showed similar death rate (APP-Veh, *n* = 3/43; WT-Veh, *n* = 1/41; APP-NSC, *n* = 2/47; [*n* = number of mice dead/total mice per group]).

We also demonstrated that hNSC transplantation affected tau phosphorylation, Aβ production, neuroinflammation, synaptic density, and cell survival in the transgenic mouse brain and identified those molecular mechanisms likely involved in improving spatial memory in these mice. It is reasonable to surmise that grafted cells exert their therapeutic potency by modifying the deleterious milieu that contributes to AD pathologies through influencing areas remote from the original cell-injection site, either by migration or release of diffusible factors. NSCs reportedly express not only a wide range of trophic factors that restrain tau phosphorylation, Aβ production, and cell death but also immunomodulatory factors [34, 41–48]. Therefore, grafted hNSCs would be expected to express these factors and interact with host cells via multiple mechanisms, resulting in the functional recovery of cognitive deficits in NSE/APPsw transgenic mice.

NSE/APPsw transgenic mice undergo a marked increase of tau phosphorylation, consistent with other AD mouse models engineered with human mutant APP [26, 49]. The hNSCs generated not only neurotrophins but also other trophic factors, including VEGF, FGF2, and GDNF, that activate Akt. Furthermore, hNSC transplantation increased the levels of neurotrophins in the transgenic mouse brain. Therefore, trophic factors augmented by hNSC transplantation prevent tau phosphorylation via Akt/GSK3β signaling because Akt activation inhibits tau phosphorylation through GSK3β inactivation [50, 51]. However, NSC transplantation does not consistently interfere with tau phosphorylation in other mouse models generated with human mutant tau, which ubiquitously enhance tau phosphorylation and develop neurofibrillary tangles [21, 52]. Although the differences among these studies are not fully understood, another study [53] and ours demonstrated that NSC transplantation perturbs tau phosphorylation induced by elevated Aβ. Alternatively, the small decrease in Aβ levels observed in the present study may have affected levels of phosphorylated tau because Aβ directly precedes aberrant tau phosphorylation [54–56].

Accumulating evidence suggests that soluble Aβ oligomers or intracellular Aβ may have a deteriorative effect on cognitive function in AD mouse models [32, 33, 57]. In the present study, NSE/APPsw transgenic mice displayed intracellular Aβ deposits rather than excessive Aβ plaques. Moreover, the production of Aβ is reduced by pharmacological or genetic inhibition of GSK3β and promoted by NGF and BDNF deprivation [46, 58–60]. Thus, trophic factors secreted from hNSCs would have helped to reduce soluble Aβ in the transgenic mouse brain and APPsw-expressing SK-N-MC cells due to down-regulated Aβ production via Akt/GSK3β signaling. We also found that the level of BACE1 was altered in the brains of hNSC-grafted transgenic mice and in hNSC-derived CM-treated APPsw-expressing cells. These results are similar to other studies reporting that BACE1 expression is decreased after activation of the phosphatidylinositol 3-kinase/Akt and mitogen-activated protein kinase/extracellular signal-regulated protein kinase pathways, or inactivation of GSK3β [34, 35]. Additionally, hNSCs do not affect the levels of *Mme* and *Ide* in brains of NSE/APPsw transgenic mice following transplantation, and do not effectively degrade soluble Aβ42 peptides *in vitro*. Therefore, the decrease of soluble intracellular Aβ in hNSC-transplanted transgenic mice is attributable to the attenuated APP processing via the Akt/GSK3β signaling-mediated reduction of BACE1 expression, not to the increased Aβ clearance. While the exact nature of Aβ formation and tau phosphorylation remains elusive, our findings strongly suggest that hNSC transplantation mitigates aberrant tau phosphorylation and intracellular Aβ accumulation through trophic factor-dependent GSK3β inactivation in NSE/APPsw transgenic mice.

Recently, some studies describe that trophic factors, especially BDNF directly control tau phosphorylation and Aβ production [46, 51]. Thus, to determine whether BDNF secreted from hNSCs is directly involved in tau phosphorylation and BACE1 expression in this study, we tested brain slices from NSE/APPsw transgenic mice with BDNF-depleted CM using immunoprecipitation. The treatment of hNSC-derived CM significantly induced the reduction of phosphorylated tau and BACE1 expression in brain slices compared with controls, whereas BDNF-depleted CM revealed no significant change in the level of tau phosphorylation and BACE1 (the Additional file 6: Figure S6). Thus, hNSC-secreted BDNF is directly involved in regulating BACE1 expression as well as tau phosphorylation. However, besides mechanistic studies using brain slices, further studies are needed to ascertain that BDNF is dominantly involved in tau phosphorylation and BACE1 expression by transplanting BDNF- as well as other factor-knockout hNSCs.

In the present study, astrogliosis and microgliosis were enhance along with elevated expression of pro-inflammatory mediators in NSE/APPsw transgenic mice, consistent with observations in individuals with AD and other mouse models of AD [3, 36]. Previous reports indicate that neuroinflammation induced by chronically activated microglia in AD mouse models exacerbates neurodegeneration, even though microglia initially phagocytose Aβ [3]. NSC transplantation markedly attenuates microgliosis and modulates immune responses through interactions of grafted NSCs with T cells, microglia, or dendritic cells in various neurological disease models [40, 42, 61–63]. In the present study, hNSC transplantation reduced astrogliosis, microgliosis and the expression of pro-inflammatory mediators in transgenic mice. Furthermore, hNSCs co-cultured with LPS-stimulated microglial cells expressed anti-inflammatory factors. We demonstrated in particular that TGF-β1, CD200, and CD47 generated from hNSCs were directly implicated in the expression of the pro-inflammatory mediators in microglia. TGF-β1, an anti-inflammatory cytokine, protects the brain against microglia-mediated neurotoxicity and enhances microglial function for Aβ clearance [64, 65]. CD200 and CD47 constitutively maintain microglia in a resting state and impede their pro-inflammatory activity [66]. Therefore, our results suggest that hNSCs exert neuroinflammatory attenuation in transgenic mice by inducing microglial deactivation. Alternatively, decreased expression of IL-1β in hNSC-transplanted transgenic mouse brains may reverse tau phosphorylation, as it was reported that the release of IL-1β from microglia stimulates tau phosphorylation [67]. Moreover, the attenuation of neuroinflammation may be associated with the reduction of BACE1 because anti-inflammatory drugs decrease BACE1 expression, leading to reduced Aβ levels [68, 69].

Through the regenerative and protective properties of NSCs, NSC grafting increases synaptic plasticity and decreases apoptotic cell death via trophic supplies [15, 19, 21, 52, 70]. Similarly, our results suggest that hNSC transplantation reinforces synaptic density and prevents cell loss in NSE/APPsw transgenic mice.

## Conclusions

This study demonstrated that human fetal brain-derived NSCs showed extensive migration, robust engraftment, long-term survival, differentiation into three CNS neural cell types, albeit that most cells remained in an immature state following transplantation. hNSC grafting not only facilitate synaptic plasticity and anti-apoptotic function via trophic supplies, but also decrease tau-phosphorylation via Trk-dependent Akt/GSK3β signaling, Aβ production via Akt/GSK3β-mediated reduced BACE1 expression, and neuroinflammation through deactivation of microglia mediated by cell-to-cell contact and secreted anti-inflammatory factors, resulting in improved spatial memory in NSE/APPsw transgenic mice. Furthermore, the safety and feasibility of hNSC transplantation are supported. There was no adverse finding after hNSC implantation into the brain of transgenic mice. Thus, hNSCs are a highly safe and effective therapeutic strategy for treating AD by modulating complex brain systems using multiple mechanisms. However, the cognitive recovery following hNSC grafting was not maintained over the long-term; thus, the long-term benefit of hNSC transplantation remains unclear. With further advances in understanding the pathology and discovering novel therapeutic targets, hNSCs genetically or non-genetically modified to release disease-modifying proteins could be used as a versatile tool, adding to the inherent benefits of hNSC transplantation in AD treatment.

## Methods

### Mice

NSE/APPsw transgenic mice were bred with background-matched C57BL/6 mice. Heterozygous transgenic mice and wild-type mice from the same litters were divided based on genotyping at 3–4 weeks of age [26] and used at 13 months of age under a protocol approved by the Institutional Animal Care and Use Committee at Yonsei University College of Medicine in Seoul, Korea. Mice were housed in groups of 4–5 under a 12 h light/dark cycle at 22 °C, fed *ad libitum*, and maintained in a facility accredited by the Association for the Assessment and Accreditation of Laboratory Animal Care International.

### Human NSC culture

Human fetal brain tissue from a cadaver at 13 weeks of gestation was obtained with full parental consent and the approval of the Research Ethics Committee of Yonsei University College of Medicine (Permit Number: 4-2003-0078). The hNSCs isolated from the telencephalon were grown as neurospheres in serum-free culture medium (DMEM/F12; Gibco, Grand Island, NY), N2 formulation (Gibco), and 8 μg/ml of heparin (Sigma, St. Louis, MO) supplemented with 20 ng/ml of FGF-2 (R&D Systems, Minneapolis, MN) and 10 ng/ml leukemia inhibitory factor (Sigma) [12]. hNSCs were maintained by passage through dissociation of bulk neurospheres and cryopreserved at each passage in a Good Manufacturing Practice facility. As previously described [38], lenti-GFP particles were prepared, and some hNSCs were transduced with a multiplicity of infection of 1 and cultured.

### Animal surgery and transplantation

At the time of grafting, hNSCs labeled with BrdU (Sigma) for 5 days were dissociated with 0.05 % trypsin-EDTA (Gibco) and washed three times with H-H buffer (pH 7.4; Gibco). The entire cell pellet was then resuspended in H-H buffer at a density of 1 × 10^5^ cells/μl. Additionally, lenti-GFP-transduced hNSCs were prepared in parallel. Mice were anesthetized with a mixture of ketamine (50 mg/kg) and xylazine (Rompun, 10 mg/kg), and then injected with 5 μl of vehicle or an hNSC suspension bilaterally into lateral ventricles (LVs; 0.1 mm caudal, 0.9 mm bilateral to bregma, and 2.0 mm ventral from the dura mater) of the brain at a flow rate of 1 μl/min using a Hamilton syringe on an infusion pump (KD Scientific, Holliston, MA). After the injection, the needle was kept in place for 5 min before it was slowly withdrawn. All mice received daily injections of cyclosporine A (10 mg/kg, intraperitoneally) from the day before transplantation to the end of the experiment.

### Behavioral analysis

The open field test was conducted for 30 min or 22 h using an infrared beam break detection system (Med Associates, St. Albans, VT) to record the numbers of ambulatory and stereotyped activities for 5 min or 2 h per session. The accelerating rotarod task measured the latencies to fall off the rod (LSi Letica; Panlab, Barcelona, Spain) that was accelerated gradually from 4 to 40 rpm for the 5 min test period. The Morris water maze test was conducted with automated video tracking software (Smart; Panlab). A mouse was placed next to and facing the wall in four individual directions with a hidden escape platform using an intertrial interval of 15 min for 6 consecutive days. After the acquisition phase, the probe trial was conducted without the platform present on day 7. The mouse was allowed to swim for 1 min. Additional details are described in the Additional file 7.

### Immunohistochemistry

At 7 weeks post-transplantation, the brains were fixed and coronally sliced into 20 μm sections as described [38]. The sections on the slide were blocked with 10 % normal donkey serum (Jackson ImmunoResearch, West Grove, PA), 3 % bovine serum albumin, and 0.3 % Triton X-100 in PBS. BrdU staining required treatment with 2 N HCl for 30 min at 37 °C prior to blocking. The sections were incubated with antibodies as described in the Additional file 8: Table S1 and visualized with fluorescein- or Texas Red-conjugated antibodies (Vector, Burlingame, CA). Alternatively, after treatment with 0.3 % H_2_O_2_ in methanol, immunolabeling for Aβ plaques required treatment with 70 % formic acid for 15 min [71]. The sections were incubated with mouse anti-Aβ1-16 (6E10, 1:200; Covance, Dedham, MA), rabbit anti-Aβ42 (1:100; Invitrogen, Carlsbad, CA) or mouse anti-PHF-tau (AT180, 1:200; Thermo Scientific, Rockford, IL) antibodies, followed by biotinylated antibodies (Jackson ImmunoResearch), and were visualized using the Vectastain Elite ABC kit according to the manufacturer’s instructions. Other sections for the TUNEL assay were subjected to an In Situ Cell Detection Kit (Roche, Mannheim, Germany) following the manufacturer’s procedures. Immunolabeled specimens were observed with an Olympus BX51 microscope and a Zeiss LSM 700 confocal microscope and analyzed with ImageJ version 1.46r software (National Institutes of Health). Additional details were provided in the Additional file 7.

### Western blot and ELISA

Mouse brains were homogenized in Tissue Protein Extraction Reagent (Thermo Scientific) containing protease and phosphatase inhibitors (Sigma) at 7 weeks post-transplantation. The homogenates were briefly sonicated and then centrifuged for 1 h at 1 × 10^5^ *g*. The pellet was subsequently re-homogenized in 70 % formic acid and centrifuged for 1 h at 1 × 10^5^ *g*. The first and second supernatants were prepared as the detergent-soluble and insoluble fractions, respectively. Samples were electrophoresed in 10 % Tris-glycine gels, 4 − 15 % Mini-protean TGX precast gels (Bio-Rad, Hercules, CA), or 16.5 % Tris-tricine gels, and transferred to nitrocellulose membranes. After being blocked with 5 % skim milk or bovine serum albumin in TBS containing 0.1 or 0.05 % Tween 20, the membranes were incubated with antibodies as described in the Additional file 8: Table S1. Next, the membranes were incubated with peroxidase-conjugated antibodies (Jackson ImmunoResearch), treated with SuperSignal West Pico or Dura chemiluminescent substrate (Thermo Scientific), and observed with an LAS-4000 mini (GE Healthcare, Wausaukee, WI). Protein bands were analyzed as the signal intensity ratio between target protein and β-actin using Multi Gauge version 3 software (Fujifilm, Tokyo, Japan). Aβ40 and Aβ42 levels were measured with ELISAs using the human Aβ1 − 40 or Aβ1 − 42 assay kit (Immuno-Biological Laboratories, Minneapolis, MN) in accordance with the manufacturer’s instructions.

### Quantitative PCR

The RNA was isolated from hNSCs, BV2 murine microglial cells, or mouse brains at 7 weeks post-transplantation using TRI Reagent solution (Molecular Research Center, Cincinnati, OH) and reverse-transcribed into cDNA using SuperScript III Reverse Transcriptase (Invitrogen). The quantitative (q)PCR was performed in 384-well plates with LightCycler 480 SYBR Green I Master mix (Roche) on a LightCycler 480 System (Roche) as follows: 95 °C for 5 min and 45 cycles of 95 °C for 10 s, 60 °C for 20 s, and 72 °C for 15 s, followed by a melting curve program. The forward and reverse primers (Additional file 9: Table S2) were designed using PrimerBank and RTPrimerDB databases (http://pga.mgh.harvard.edu/primerbank/index.html and http://www.rtprimerdb.org). The relative gene expression was analyzed using advanced relative quantification based on the E-method provided by Roche Applied Science. Expression levels were normalized against *Gapdh* or 18S rRNA with a PCR efficiency correction.

### Treatment of conditioned media

The CM from hNSCs and fibroblasts were concentrated 10-fold using Amicon Ultra-0.5 centrifugal filter devices (Millipore, Milford, MA), according to the manufacturer’s manual. More details were described in the Supporting Information. Differentiated PC12 cells (4 × 10^5^) [30] on six-well plastes were treated with 2 μM soluble Aβ42 (Invitrogen) in the presence of concentrated CM in RPMI 1640 medium (Gibco) for 24 h. Soluble Aβ42 was prepared as previously described [72]. APPsw-expressing SK-N-MC cells (2 × 10^5^) [38] were seeded on six-well plates in growth medium, and then the medium was completely exchanged for fresh DMEM the following day in the presence of concentrated CM. The cultured media were immunoprecipitated with 4 μg of anti-6E10 after 24 h, using 20 μl of Dynabead ProteinG (Invitrogen) according to the manufacturer’s protocol to estimate Aβ content. The cells were lysed in RIPA buffer (Thermo Scientific) with Halt Protease and Phosphatase Inhibitor Cocktail (Thermo Scientific) for western blot.

### Co-culture of hNSCs and BV2 cells

The hNSCs (1.2 × 10^6^) were differentiated on PLL-coated six-well plates (lower chamber) for 3 days and were then co-cultured with BV2 microglial cells (1.2 × 10^6^) on the 0.4 μm porous inserts (upper chamber) of Transwell permeable supports (Corning, Corning, NY) in serum-free culture medium with added LPS for 24 h. Additionally, hNSCs (2 × 10^6^) differentiated on PLL-coated 6 cm dishes for 5 days were directly co-cultured with BV2 cells (2 × 10^6^) in the presence of LPS. Mixed cultures of hNSCs and BV2 cells were separated using fluorescence-activated cell sorting (FACS; FACSAria II; sort nozzle, 100 μm, and sheath pressure, 20 psi) after 24 h. The hNSCs and BV2 cells were dissolved in TRI reagent.

### Transfection of small interfering RNA

The BV2 microglial cells were transfected with 10 μM *Tgfbr2* small interfering RNA (siRNA) (sense, 5′ CAGAAGAUGGCUCGCUGAAdTdT 3′; antisense, 5′ UUCAGCGAGCCAUCUUCUGdTdT 3′), *Sirpa* siRNA (sense, 5′ CUCUACCCAACUUGAGCUUdTdT 3′; antisense, 5′ AAGCUCAAGUUGGGUAGAGdTdT 3′), *Cd200r1* siRNA (sense, 5′ CUCUAUGAUACUGUGACUAdTdT 3′; antisense, 5′ UAGUCACAGUAUCAUAGAGdTdT 3′), or non-functioning negative-control siRNA (sense, 5′ CCUACGCCACCAAUUUCGUdTdT 3′; antisense, 5′ ACGAAAUUGGUGGCGUAGGdTdT 3′) using Lipofectamine RNAiMAX reagent (Invitrogen) according to the manufacturer’s protocols. All siRNAs were purchased from Bioneer (Daejeon, Korea). After 3 h of siRNA lipofection in these co-cultures, LPS was added to the cultures. The BV2 cells were separated from the mixed cultures after a 24 h LPS treatment and used for qPCR.

### Statistical analysis

Statistical analyses were conducted using SPSS version 20 (IBM Corp., Armonk, NY) software. Behavioral values were subjected to repeated-measures ANOVA or one-way ANOVA, followed by *post hoc* Scheffé’s tests. Differences between two groups were assessed using the Mann–Whitney *U*-test as a non-parametric analysis. Data are presented as means ± standard error of the means (SEM), error bars indicate ± SEM, and *p*-values <0.05 were considered statistically significant.

## Additional files

Additional file 1: Figure S1. GFP expression by lenti-GFP-transduced hNSCs. Proliferating lenti-GFP-transduced hNSCs form neurospheres in culture dishes (A) and express GFP (B). Flow cytometry analysis using FlowJo (version 9.3.3) software showed that 94.5 % of all cells are GFP-positive (blue line histogram in C). The red line histogram is the negative control. Scale bar, 100 μm. (TIFF 310 kb) Additional file 2: Figure S2. Histological analysis of transplanted hNSCs into NSE/APPsw transgenic mice at 3 months post-grafts. (A–D) Some grafted hNuC^+^ and human cytoplasmic marker SC121^+^ cells are found in the subventricular zone (A and B), third ventricle (C), and hypothalamus (D). Z-stack images are built and compiled to maximal intensity projections. Scale bars, 50 μm (A-D). (E–G) Most grafted hNuC^+^ (red) cells expressed hnestin (green; E) whereas some cells are co-localized with either TUJ1 (green; F) or GFAP (green; G). Scale bars, 25 μm (E-G). (H) A few of SC121^+^ (red) cells express Olig2. Scale bars, 25 μm. In E–H, confocal image stacks are orthogonally presented as x–z (bottom) and y–z (right) planes indicated by the vertical green and horizontal red lines, respectively. (TIFF 5979 kb) Additional file 3: Figure S3. hNSCs express diverse trophic factors. (A) *In vitro* proliferating and differentiated hNSCs expressed *BDNF*, *NTF3*, *NTF4*, *NGF*, *VEGF*, *FGF2*, *and GDNF*. (B) Western blotting analysis showed that hNSCs secreted higher levels of BDNF, NTF3, NTF4, NGF, and VEGF into the culture medium than human foreskin fibroblasts secrete. (TIFF 127 kb) Additional file 4: Figure S4. The expression of Aβ-degrading enzymes in both *in vivo* and *in vitro*. (A) Transplantation of hNSCs (NSC, *n* = 7) did not significantly alter the levels of *Mme* and *Ide* expression in NSE/APPsw transgenic mice compared with vehicle injection (Veh, *n* = 6). (B) *In vitro* expression of Aβ-degrading enzymes in hNSCs. hNSCs under proliferation and differentiation conditions expressed *IDE*, *MME*, *ECE1* (endothelin converting enzyme 1), *ECE2* (endothelin converting enzyme 2), *MMP2* (matrix metalloproteinase 2), *PLAT* (plasminogen activator, tissue), *PLAU* (plasminogen activator, urokinase), *ACE* (angiotensin 1 converting enzyme), and *CTSB* (cathepsin B). On western blot, there were no differences in the levels of Aβ42 in the media containing 1 μM soluble Aβ42 peptides between wells with and without incubation with hNSCs for 2 days. The number of mice (n) in A is indicated. All data represent mean ± SEM. Error bars indicate ± SEM. (TIFF 3170 kb) Additional file 5: Figure S5. The separation of mixed co-cultured hNSCs and BV2 microglial cells. (A) Representative images of human NSCs and lipopolysaccharide (LPS)-activated BV2 cells separated by flow cytometry from a mixed co-culture. Human NSCs and LPS-activated BV2 cells were sorted based on different combinations of forward scatter-A (FSC-A, cell size) and side scatter-A (SSC-A, granularity). The percentage of hNSCs and BV2 cells is 18.6 and 71.6 %, respectively, in the co-culture of hNSCs (FSC-A^lo^/SSC-A^lo^) and LPS-activated BV2 cells (FSC-A^hi^/SSC-A^hi^). The sorted hNSCs and LPS-activated BV2 cells are rarely included with other cells (<3 %). (B) The siRNA-lipofected BV2 cells decrease target gene expression (>50 %; *n* = 3 per group). The number of experiments (n) is indicated. All data represent mean ± SEM. All error bars indicate SEM. Mann–Whitney *U*-test, **p* < 0.05. (TIFF 527 kb) Additional file 6: Figure S6. NSE/APPsw transgenic mouse-derived brain slices treated with BDNF-depleted CM. (A) Western blot showed that hNSCs secreted high level of BDNF into the cultured medium (lane 1; CM), and anti-BDNF antibody-mediated immunoprecipitation effectively removed BDNF in CM (lane 2; BDNF-depleted CM). Another SDS-PAGE gel was in parallel performed with silver staining to verify even loading. (B) Brain slices treated with DMEM (Ctr), CM, BDNF-depleted CM. Western blot analysis of phosphorylated tau (AT180) and BACE1 (*n* = 3 per group, where n is the number of experiments). All data represent mean ± SEM. Error bars indicate ± SEM. Mann–Whitney *U*-test, **p* < 0.05. (TIFF 2110 kb) Additional file 7:
**Additional details of Methods.** (DOCX 33 kb) Additional file 8: Table S1. List of primary antibodies used in immunohistochemistry (IHC) and western blot (WB). (DOCX 28 kb) Additional file 9: Table S2. Sequences of primers used for reverse transcription (RT) and quantitative (q) PCR. (DOCX 25 kb)

## Abbreviations

AD: Alzheimer’s disease
Aβ: Amyloid-β
hNSCs: Human neural stem cells
NSE: Neuron-specific enolase
GSK3β: Glycogen synthase kinase 3β
CM: Conditioned media
