# Supplementary figures and images for: Human neural stem cells alleviate Alzheimer-like pathology in a mouse model
Source: Mol Neurodegener. 2015 Aug 21;10:38. doi: 10.1186/s13024-015-0035-6 (PMC4546205; doi:10.1186/s13024-015-0035-6)

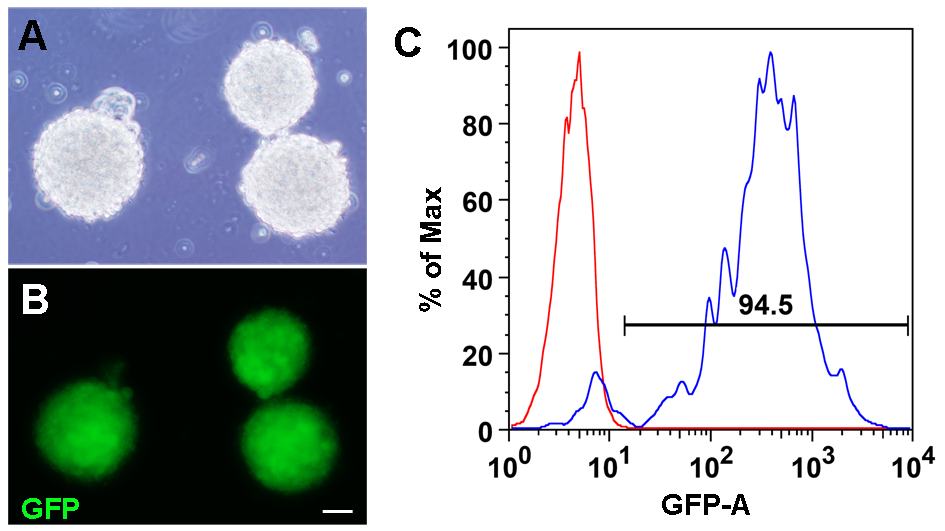

Supplement: Additional file 1: Figure S1. — GFP expression by lenti-GFP-transduced hNSCs. Proliferating lenti-GFP-transduced hNSCs form neurospheres in culture dishes (A) and express GFP (B). Flow cytometry analysis using FlowJo (version 9.3.3) software showed that 94.5 % of all cells are GFP-positive (blue line histogram in C). The red line histogram is the negative control. Scale bar, 100 μm. (TIFF 310 kb) [file 13024_2015_35_MOESM1_ESM.tiff]

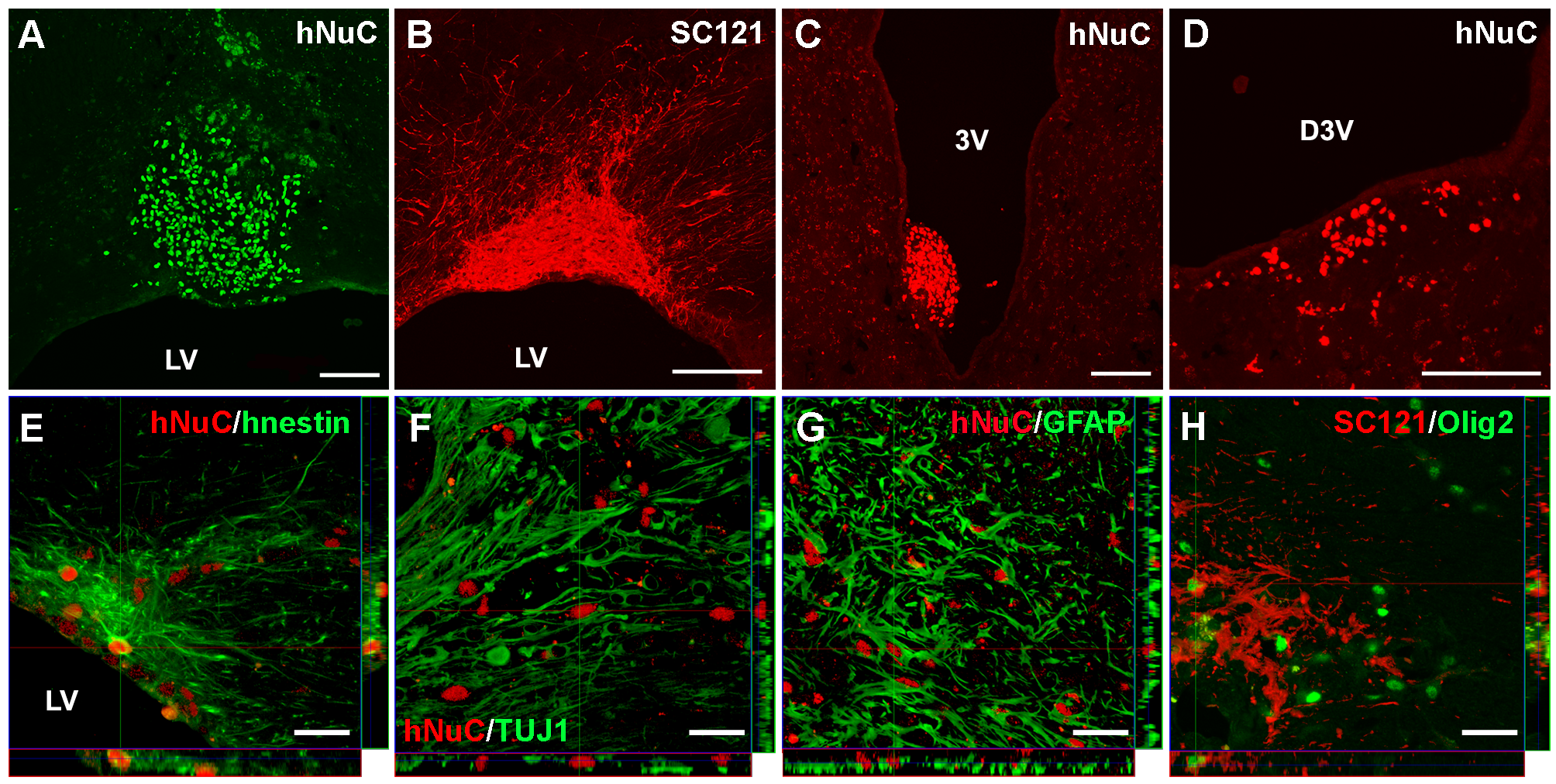

Supplement: Additional file 2: Figure S2. — Histological analysis of transplanted hNSCs into NSE/APPsw transgenic mice at 3 months post-grafts. (A–D) Some grafted hNuC+ and human cytoplasmic marker SC121+ cells are found in the subventricular zone (A and B), third ventricle (C), and hypothalamus (D). Z-stack images are built and compiled to maximal intensity projections. Scale bars, 50 μm (A-D). (E–G) Most grafted hNuC+ (red) cells expressed hnestin (green; E) whereas some cells are co-localized with either TUJ1 (green; F) or GFAP (green; G). Scale bars, 25 μm (E-G). (H) A few of SC121+ (red) cells express Olig2. Scale bars, 25 μm. In E–H, confocal image stacks are orthogonally presented as x–z (bottom) and y–z (right) planes indicated by the vertical green and horizontal red lines, respectively. (TIFF 5979 kb) [file 13024_2015_35_MOESM2_ESM.tiff]

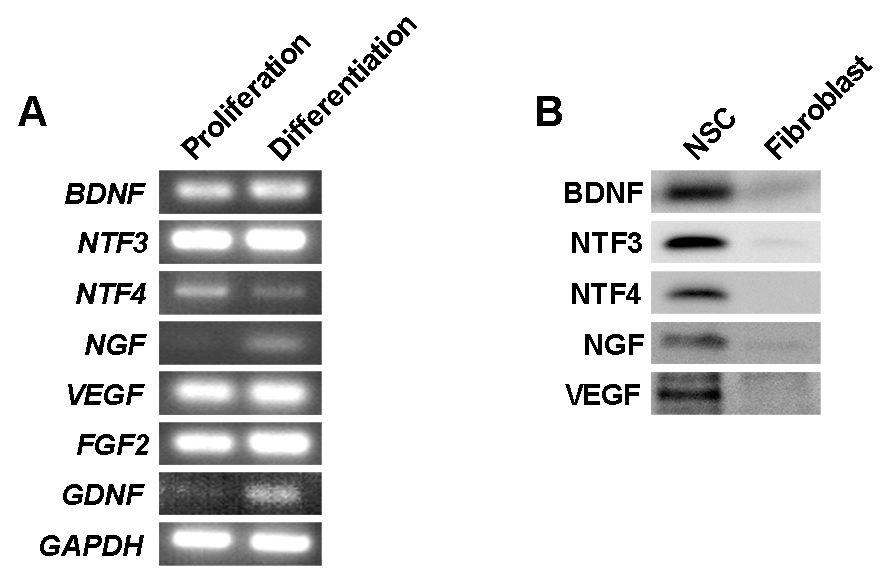

Supplement: Additional file 3: Figure S3. — hNSCs express diverse trophic factors. (A) In vitro proliferating and differentiated hNSCs expressed BDNF, NTF3, NTF4, NGF, VEGF, FGF2, and GDNF. (B) Western blotting analysis showed that hNSCs secreted higher levels of BDNF, NTF3, NTF4, NGF, and VEGF into the culture medium than human foreskin fibroblasts secrete. (TIFF 127 kb) [file 13024_2015_35_MOESM3_ESM.tiff]

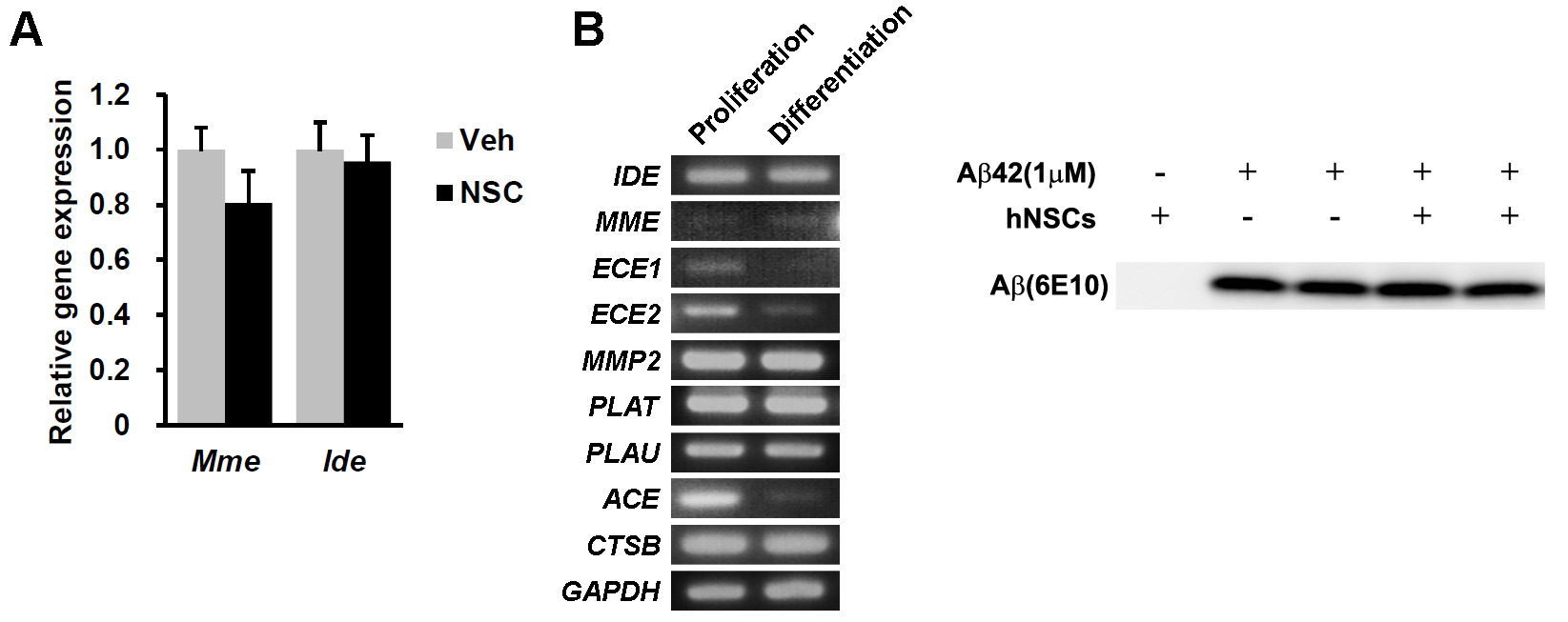

Supplement: Additional file 4: Figure S4. — The expression of Aβ-degrading enzymes in both in vivo and in vitro. (A) Transplantation of hNSCs (NSC, n = 7) did not significantly alter the levels of Mme and Ide expression in NSE/APPsw transgenic mice compared with vehicle injection (Veh, n = 6). (B) In vitro expression of Aβ-degrading enzymes in hNSCs. hNSCs under proliferation and differentiation conditions expressed IDE, MME, ECE1 (endothelin converting enzyme 1), ECE2 (endothelin converting enzyme 2), MMP2 (matrix metalloproteinase 2), PLAT (plasminogen activator, tissue), PLAU (plasminogen activator, urokinase), ACE (angiotensin 1 converting enzyme), and CTSB (cathepsin B). On western blot, there were no differences in the levels of Aβ42 in the media containing 1 μM soluble Aβ42 peptides between wells with and without incubation with hNSCs for 2 days. The number of mice (n) in A is indicated. All data represent mean ± SEM. Error bars indicate ± SEM. (TIFF 3170 kb) [file 13024_2015_35_MOESM4_ESM.tiff]

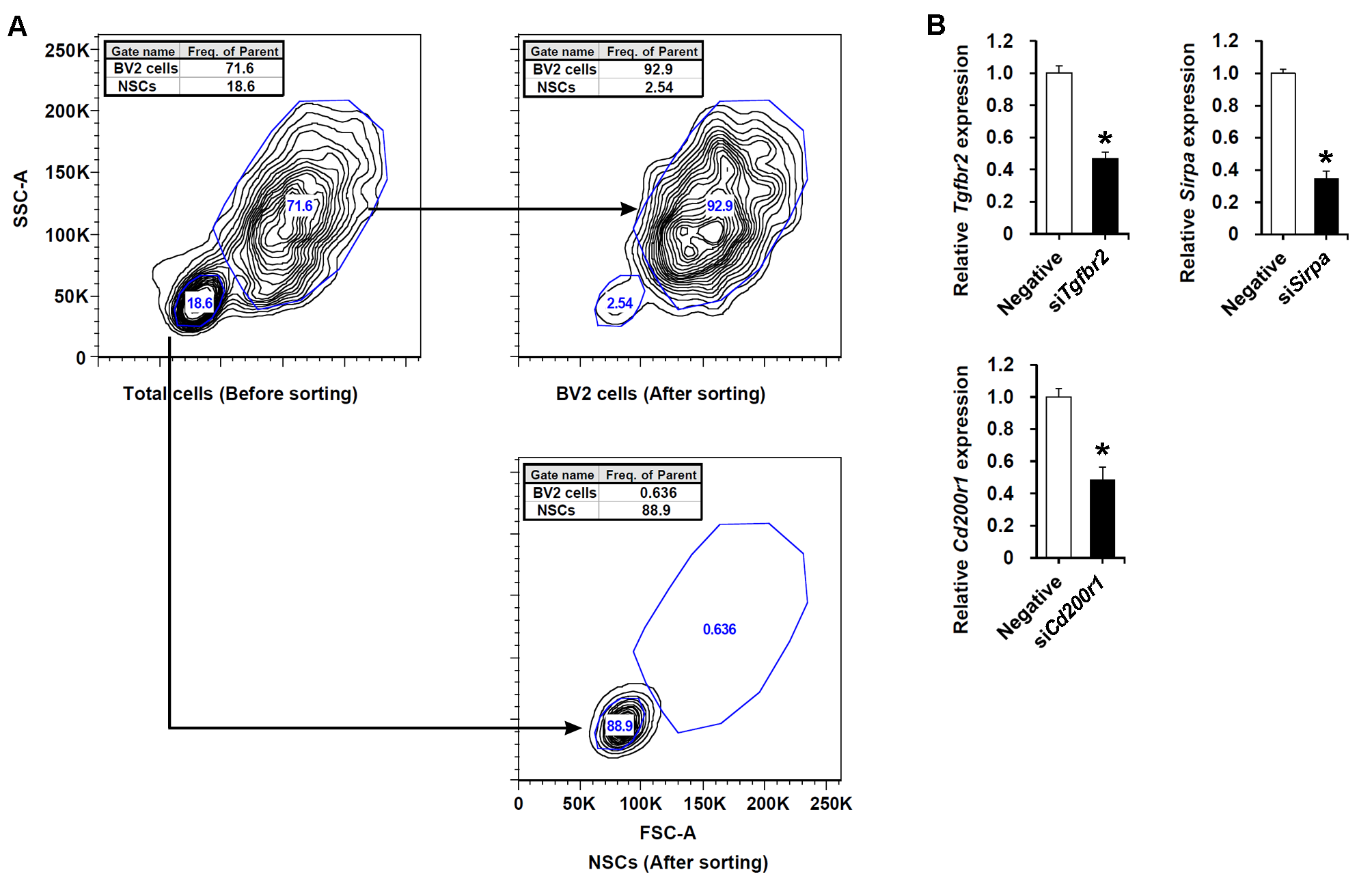

Supplement: Additional file 5: Figure S5. — The separation of mixed co-cultured hNSCs and BV2 microglial cells. (A) Representative images of human NSCs and lipopolysaccharide (LPS)-activated BV2 cells separated by flow cytometry from a mixed co-culture. Human NSCs and LPS-activated BV2 cells were sorted based on different combinations of forward scatter-A (FSC-A, cell size) and side scatter-A (SSC-A, granularity). The percentage of hNSCs and BV2 cells is 18.6 and 71.6 %, respectively, in the co-culture of hNSCs (FSC-Alo/SSC-Alo) and LPS-activated BV2 cells (FSC-Ahi/SSC-Ahi). The sorted hNSCs and LPS-activated BV2 cells are rarely included with other cells (<3 %). (B) The siRNA-lipofected BV2 cells decrease target gene expression (>50 %; n = 3 per group). The number of experiments (n) is indicated. All data represent mean ± SEM. All error bars indicate SEM. Mann–Whitney U-test, *p < 0.05. (TIFF 527 kb) [file 13024_2015_35_MOESM5_ESM.tiff]

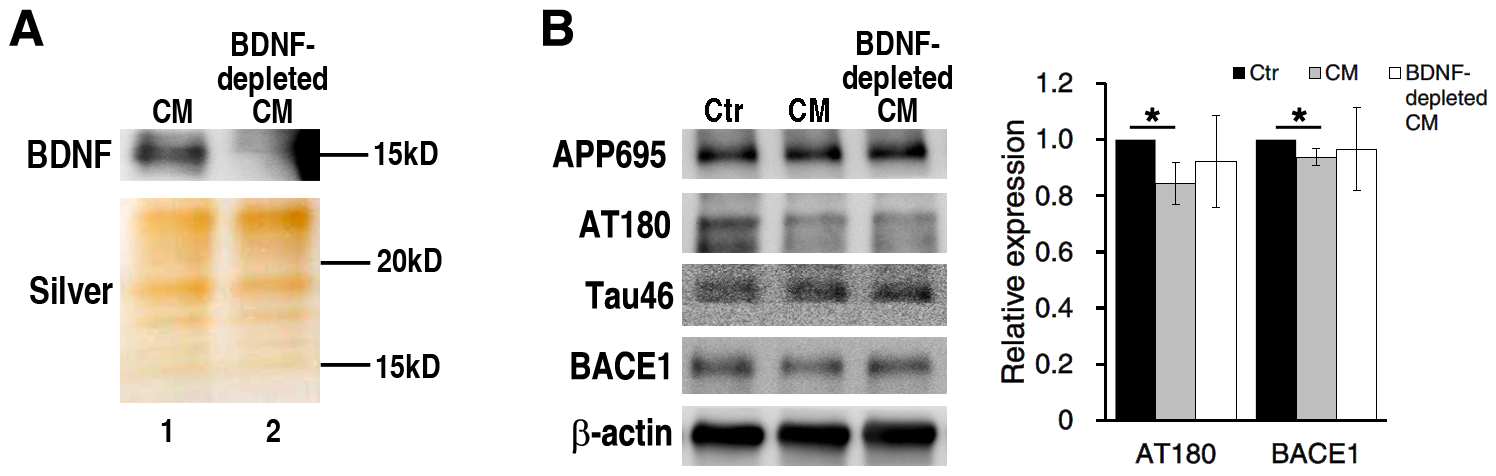

Supplement: Additional file 6: Figure S6. — NSE/APPsw transgenic mouse-derived brain slices treated with BDNF-depleted CM. (A) Western blot showed that hNSCs secreted high level of BDNF into the cultured medium (lane 1; CM), and anti-BDNF antibody-mediated immunoprecipitation effectively removed BDNF in CM (lane 2; BDNF-depleted CM). Another SDS-PAGE gel was in parallel performed with silver staining to verify even loading. (B) Brain slices treated with DMEM (Ctr), CM, BDNF-depleted CM. Western blot analysis of phosphorylated tau (AT180) and BACE1 (n = 3 per group, where n is the number of experiments). All data represent mean ± SEM. Error bars indicate ± SEM. Mann–Whitney U-test, *p < 0.05. (TIFF 2110 kb) [file 13024_2015_35_MOESM6_ESM.tiff]
