# Supplementary material for: Human neural stem cells alleviate Alzheimer-like pathology in a mouse model
Source: Mol Neurodegener. 2015 Aug 21;10:38. doi: 10.1186/s13024-015-0035-6 (PMC4546205; doi:10.1186/s13024-015-0035-6)
Supplement: Additional file 7: — Additional details of Methods. (DOCX 33 kb) [file 13024_2015_35_MOESM7_ESM.docx]

**Additional details of Methods**

**Behavioral analysis**

The open field test was conducted in a chamber made of acrylic boards (50 × 50 × 30 cm) for 30 min or 22 h using an infrared beam break detection system (Med Associates, St. Albans, VT). After the mouse was gently placed in the center of the chamber floor, it was allowed to freely explore, and the numbers of ambulatory and stereotyped activities were recorded continuously for 5 min or 2 h per session. Ambulatory activity counted bouts of successive beam breaks, while stereotyped activity measured bouts of repetitive beam breaks that occurred whenever a mouse swayed from one side to the other or engaged in scratching or grooming movements.

The accelerating rotarod task was performed by mice placed on a rod (diameter: 3 cm, width: 6 cm; LSi Letica; Panlab, Barcelona, Spain). After each mouse was placed on top of the already revolving beam (4 rpm) in an orientation opposite to the rotation of the beam and familiarized with the task, the revolution speed of the rotarod was accelerated gradually from 4 to 40 rpm for the 5 min test period. The latencies to falling off the rod were measured, with an intertrial interval of 30 min after every third trial.

The Morris water maze test was conducted in a round tank (diameter: 90 cm, wall height: 30 cm), filled with water (22 ± 1°C) to a depth of 20 cm, and performance was monitored with automated video tracking software (Smart; Panlab). A mouse was placed next to and facing the wall in successive north, east, south, and west positions. The escape platform was hidden 1 cm beneath the water surface in the middle of the northwest quadrant. The intertrial interval was 15 min, and mice were tested for 6 consecutive days. Each trial was terminated immediately when the mouse located the platform. If a mouse failed to reach the platform within 1 min, it was manually placed on the platform for 30 s. After the acquisition phase, the probe trial was conducted without the platform present on day 7. The mouse was placed next to and facing the southeastern wall and allowed to swim for 1 min. Target quadrant occupancy, meaning the time spent in the quadrant in which the platform had been located during the acquisition phase, and platform crossing, which was the number of passes through the zone in which the platform had been located, were measured.

The open field test and the accelerating rotarod task were performed at 5 weeks, and the water maze test was performed at 6 or 12 weeks after injecting hNSCs or vehicle. All apparatuses were thoroughly cleaned with 70% ethanol before each task to avoid possible bias due to odors or residues left by a previously tested mouse.

**Tissue processing**

Deeply anesthetized mice were transcardially perfused and fixed with cold phosphate-buffered saline (PBS) followed by 4% paraformaldehyde (Sigma), at 7 weeks post-transplantation. Their brains were removed, post-fixed overnight, transferred to 30% sucrose in PBS, and frozen in O.C.T. compound (Sakura Finetek, Torrance, CA). The brains were coronally sliced into 20 μm sections using a cryostat (Leica CM 1850; Leica Microsystems, Chicago, IL).

**Microscopic analysis of hNSC fate in NSE/APPsw transgenic mice**

The fate of the hNSCs in NSE/APPsw transgenic mice at 7 weeks post-transplantation was determined with unbiased bilateral counts of the number of hNuMA^+^ cells expressing hnestin, TUJ1, GFAP, or Olig2 using double-labeled immunofluorescence images captured through a Zeiss LSM 700 confocal microscope (Carl Zeiss, Jena, Germany) with a 40× objective using *Z*-scanning. At least three different regions were counted, and the results of each region were averaged for each mouse.

**Quantification of AT180 and Aβ42 immunoreactivity**

The optical density values from every sixth section of a series obtained throughout the cortex and hippocampus of each mouse were measured using ImageJ version 1.46r software (National Institutes of Health). Two areas of the stratum radiatum of the CA1 region of the dorsal hippocampus and four areas of the posterior parietal cortex were examined for AT180 immunoreactivity. Aβ42 immunoreactivity was similarly measured in one area of the CA1 pyramidal neurons of the dorsal hippocampus and two areas of the posterior parietal cortex. To determine the degree of staining using ImageJ, captured images under identical exposures (shutter speed, ISO, and aperture settings) using an Olympus BX51 microscope (Olympus, Tokyo, Japan) with a 20× objective were transformed to 8-bit gray scale and inverted to assign a value of 0 to a white pixel and a value of 255 to a black pixel. The optical density values on each section were normalized to the value obtained from level-matched another section on the same slide and this section had been processed without primary antibodies to determine the cut-off threshold values. The average of the individual measurements was used to calculate group means, and either AT180 or Aβ42 levels were presented as relative optical densities in each brain region of interest in hNSC- or vehicle-injected NSE/APPsw transgenic mice. The relative immunoreactivity was displayed after normalizing to the mean value in vehicle-injected NSE/APPsw transgenic mice.

**Quantification of Aβ plaques**

Every sixth section of a series encompassing the cortex and hippocampus was stained with 6E10 antibodies, and images were captured using the dotSlide imaging system with an Olympus BX61VS microscope, a 10× objective, a motorized stage (Märzhäuser; Wetzlar, Germany) for upright microscopes, a charge-coupled device (CCD) digital camera (Pike F505C, Allied Vision Technologies, Newburyport, MA), an SL50 automated slide loader, dotSlide software, and OlyVIA software (Olympus). The total area and numbers of amyloid plaques in each mouse brain were measured with ImageJ. After an intensity threshold level was manually set to discriminate between plaque immunoreactivity and background labeling, the threshold was kept constant. Group means of plaque numbers and areas were based on the sum of the counts from each mouse.

**Quantification of GFAP, synaptophysin, and PSD95 immunoreactivity**

Every sixth section of a series taken throughout the hippocampus and cortex was labeled with GFAP, synaptophysin, or PSD95. The resulting immunofluorescence images were captured using an Olympus BX51 microscope or a Zeiss LSM 700 confocal microscope with 10× (GFAP) and 40× (synaptophysin and PSD95) objectives under respective identical settings (shutter speed, ISO, and aperture). These captured images were transformed to 8-bit gray scale using ImageJ. The optical density in regions containing the stratum radiatum of the CA1 and the polymorph layer of the dentate gyrus or the posterior parietal cortex was quantified. To normalize the optical density measurements in each section, the white matter provided background levels in the same section were subtracted from the region of interest measurements. The mean of pixel intensity per mouse was averaged, and these values were compared between hNSC- and vehicle-injected NSE/APPsw transgenic mice. The relative immunoreactivity was displayed after normalizing to the mean value in vehicle-injected NSE/APPsw transgenic mice.

**Quantification of Iba1 immunoreactivity**

Every sixth section of a series taken throughout the cortex and hippocampus of each mouse was stained with Iba1 antibodies. Each image was captured with an Olympus BX51 microscope using a 20× (cortex) or 40× (hippocampus) objective. Two areas each from the dorsal hippocampus and the posterior parietal cortex were selected in each mouse. Iba1 immunoreactivity was quantified using ImageJ as a percent of the area fraction covered by Iba1 labeling and determined as a relative value. The images were transformed to 8-bit gray scale, inverted, and then converted into binary (black and white) images by applying a threshold value that kept the Iba1-immunopositive structures without the background staining. The average of the individual measurements was used to process group means. Relative Iba1 immunoreactivity was displayed after normalizing to the mean value in vehicle-injected NSE/APPsw transgenic mice.

**TUNEL assay**

The sections for the terminal deoxynucleotidyl transferase dUTP nick-end labeling (TUNEL) were quenched in 3% H_2_O_2_ in methanol for 10 min and then permeabilized with 0.1% Triton X-100 in freshly prepared 0.1% sodium citrate buffer. After blocking with 10% normal donkey serum in PBS, these sections were subjected to an In Situ Cell Death Detection Kit (Roche) using the manufacturer’s recommended procedures. Every sixth section in a series taken throughout the cortex was analyzed for TUNEL^+^ cells using an Olympus BX51 microscope with a 40× objective. Cell counts were performed manually on each captured field.

**cDNA synthesis and reverse transcription PCR**

RNA quantity was determined spectrophotometrically, and 4 μg of isolated RNA were reverse-transcribed using SuperScript III Reverse Transcriptase (Invitrogen). Reverse transcription PCR was conducted in a 20 μl reaction mixture containing 1 μl of cDNA and using the following cycle parameters: 30 s at 95°C, 30 s at 53°C, and 30 s at 72°C for 31 cycles. Forward and reverse primers were designed to evaluate the expression levels of trophic factors and anti-inflammatory mediators in hNSCs (Supporting Information Table S1).

**Preparation of conditioned media**

The hNSCs (8 × 10^6^ cells) were plated on poly-L-lysine (PLL)-coated 10 cm culture dishes in 10 ml of serum-free culture medium and differentiated for 3 days. To prepare hNSC-conditioned medium (CM), whole medium was replaced with fresh RPMI 1640 medium (Gibco) or DMEM (Gibco), and incubated for 24 h. Alternatively, human foreskin fibroblast-CM was prepared at a density of 6 × 10^6^ cells under the same conditions, in parallel. These CMs were harvested, cleared by centrifugation at 1,650 *g* for 5 min at the end of this period, and stored at −70°C until next analysis.

**Brain slice culture**

NSE/APPsw transgenic mouse brains at 15 months old were extracted, embedded in 3% agarose, and coronally cut on a vibratome (Motorized Advance Vibroslice MA752; Campden Instrument, Lafayette, IN) in artificial CSF (125 mM NaCl, 2.5 mM KCl, 1 mM MgCl2 ,2mM CaCl2 , 1.25 mM NaH2PO4 , 25 mM NaHCO3 , 25 mM D-(+)-glucose). Three to four 300-μm-thick slices throughout the cortex and hippocampus were placed on Milli-Cell membrane inserts (0.4-μm pore size, 30-mm diameter; Millipore) in 6-well plates, and immersed in slice culture medium (66% DMEM, 25% Hanks’ balanced salt solution, 5% FBS, 1% N-2 supplement, 1% Penicillin-Streptomycin, 1% glutamine, 0.66% D-(+)-glucose) for 24 h. CM was obtained after differentiated hNSCs were incubated with DMEM for 24 h. To remove BDNF in CM, 30-μl Dynabeads ProteinG (Invitrogen) were incubated with 4-μg rabbit anti-BDNF in PBS containing 0.02% Tween-20 at room temperature for 10 min according to the manufacturer’s instructions. CM was added to anti-BDNF antibodies-coated Dynabeads and then incubated at room temperature for 30 min. BDNF-immunodepleted CM was separated from the Dynabeads-antibodies-antigens complex on the magnet. Brain slices were washed with PBS, and treated with BDNF-depleted CM, CM, and DMEM as control. After 24 h, these slices were lysed in RIPA buffer with protease and phosphatase inhibitors for western blot. These experiments were independently evaluated in triplicate.
