# Supplementary material for: Human neural stem cells alleviate Alzheimer-like pathology in a mouse model
Source: Mol Neurodegener. 2015 Aug 21;10:38. doi: 10.1186/s13024-015-0035-6 (PMC4546205; doi:10.1186/s13024-015-0035-6)
Supplement: Additional file 8: Table S1. — List of primary antibodies used in immunohistochemistry (IHC) and western blot (WB). (DOCX 28 kb) [file 13024_2015_35_MOESM8_ESM.docx]

Table S1: List of primary antibodies used in immunohistochemistry (IHC) and western blot (WB).

|  | Antibody | Species | Dilution | Company | Cat. No. |
| --- | --- | --- | --- | --- | --- |
| IHC | BrdU-fluorescein | Mouse | 1/20 | Roche | 11202693001 |
|  | Human nuclei | Mouse | 1/100 | Millipore | MAB4383 |
|  | Human nuclear matrix | Mouse | 1/25 | Calbiochem | NA09L |
|  | GFP | Rabbit | 1/200 | Invitrogen | A11122 |
|  | TUJ1 | Rabbit | 1/1000 | Covance | PRB-435P |
|  | Doublecortin | Goat | 1/200 | Santa Cruz Biotech | sc-8066 |
|  | GFAP | Rabbit | 1/1500 | Dako | Z0334 |
|  | PDGFR-α | Rabbit | 1/100 | Santa Cruz Biotech | sc-338 |
|  | Olig2 | Rabbit | 1/500 | Millipore | AB9610 |
|  | Human nestin | Rabbit | 1/200 | Millipore | ABD69 |
|  | CD11b | Rat | 1/50 | AbD serotec | MCA74G |
|  | F4/80 | Rat | 1/50 | AbD serotec | MCA497GA |
|  | Iba1 | Rabbit | 1/250 | Wako | 01919741 |
|  | Synaptophysin | Mouse | 1/100 | Sigma | S5768 |
|  | PSD95 | Rabbit | 1/50 | Cell signaling | 2507 |
| WB | APP695 | Mouse | 1/1000 | Zymed | 130200 |
|  | Βeta amyloid (6E10) | Mouse | 1/1000 | Covance | SIG39320 |
|  | Tau | Mouse | 1/1000 | Sigma | T5530 |
|  | Tau (Tau46) | Mouse | 1/1000 | Cell signaling | 4019 |
|  | p-Tau (Ser404) | Rabbit | 1/2000 | Santa Cruz Biotech | sc-12952-R |
|  | PHF-Tau (AT8) | Mouse | 1/1000 | Thermo Scientific | MN1020 |
|  | PHF-Tau (AT180) | Mouse | 1/1000 | Thermo Scientific | MN1040 |
|  | Phospho-Tau (PHF13) | Mouse | 1/1000 | Cell signaling | 9632 |
|  | Trk (pan) | Rabbit | 1/1000 | Cell signaling | 4609 |
|  | Phospho-TrkA/B | Rabbit | 1/1000 | Cell signaling | 4621 |
|  | Akt (pan) | Rabbit | 1/1000 | Cell signaling | 4691 |
|  | Phospho-Akt | Rabbit | 1/1000 | Cell signaling | 13038 |
|  | GSK3β | Mouse | 1/1000 | Santa Cruz Biotech | sc-81462 |
|  | Phospho-GSK3β | Rabbit | 1/1000 | Cell signaling | 9336 |
|  | Synaptophysin | Mouse | 1/1000 | Sigma | S5768 |
|  | BACE | Rabbit | 1/1000 | Cell signaling | 5606 |
|  | APP C-Terminal | Rabbit | 1/1000 | Sigma | A8717 |
|  | β-actin | Mouse | 1/2500 | Sigma | A1978 |
|  | NTF3 | Rabbit | 1/1000 | Santa Cruz Biotech | sc-547 |
|  | NGF | Rabbit | 1/1000 | Cell signaling | 2046 |
|  | NTF4 | Rabbit | 1/1000 | Santa Cruz Biotech | sc-545 |
|  | BDNF | Rabbit | 1/1000 | Santa Cruz Biotech | sc-20981 |
|  | VEGF | Rabbit | 1/1000 | BD Pharmigen | 555036 |
|  | Active Caspase3 | Rabbit | 1/1000 | BD Pharmigen | 559565 |
|  | Synaptophysin | Mouse | 1/1000 | Sigma | S5768 |
|  | PSD95 | Rabbit | 1/1000 | Cell signaling | 2507 |
