# Supplementary material for: Human neural stem cells alleviate Alzheimer-like pathology in a mouse model
Source: Mol Neurodegener. 2015 Aug 21;10:38. doi: 10.1186/s13024-015-0035-6 (PMC4546205; doi:10.1186/s13024-015-0035-6)
Supplement: Additional file 9: Table S2. — Sequences of primers used for reverse transcription (RT) and quantitative (q) PCR. (DOCX 25 kb) [file 13024_2015_35_MOESM9_ESM.docx]

Table S2: Sequences of primers used for reverse transcription (RT) and quantitative (q) PCR.

|  | Gene name | Forward primer | Reverse primer |
| --- | --- | --- | --- |
| RT  PCR | *GAPDH* | ACCACAGTCCATGCCATCAC | TCCACCACCCTGTTGCTGTA |
|  | *BDNF* | AACAATAAGGACGCAGACTT | TGCAGTCTTTTTGTCTGCCG |
|  | *NTF3* | TACGCGGAGCATAAGAGTCAC | GGCACACACACAGGACGTGTC |
|  | *NTF4* | CCTCCCCATCCTCCTCCTTTT | ACTCGCTGGTGCAGTTTCGCT |
|  | *VEGFA* | CCATGGCAGAAGGAGGAGG | ATTGGATGGCAGTAGCTGCG |
|  | *GDNF* | CTGACTTGGGTCTGGGCTATG | TTGTCACTCACCAGCCTTCTATT |
|  | *FGF2* | GTGTGCTAACCGTACCTGGC | CTGGTGATTTCCTTGACCGG |
|  | *NGF* | ATGTCCATGTTGTTCTACACT | AAGTCCAGATCCTGAGTGTCT |
|  | *TGFB1* | TGGACATCAACGGGTTCACT | TGTCCAGGCTCCAAATGTAG |
|  | *IL4* | AAGGAAACCTTCTGCAGGGCT | CGTACTCTGGTTGGCTTCCTT |
|  | *IL13* | GAGGAGCTGGTCAACATCAC | CCTTTACAAACTGGGCCACC |
|  | *CX3CL1* | AGAGGAGAATGCTCCGTCTGA | TCTGGTAGGTGAACATGGCCA |
|  | *CD47* | TCTTTTCGTCCCAGGTGAAT | TTGGAAGCCACAAATTTCAT |
|  | *CD200* | CCTAAATATCACTTGCTCTG | ATTGAGATTAGGACGAGAAG |
|  | *IDE* | TACCGGCTAGCGTGGCTTCT | TCCTGCATGCTCACTGAGAA |
|  | *MME* | TTTAAGGAGCAATCCCAGTGC | CCTGGACTGTGCACATCTGTT |
|  | *ECE1* | AAGGCCGATGCCATCTACAAC | TTTAAGGCCTTGGGTGAGGAG |
|  | *ECE2* | CAGACCTGCATCTCCAACACG | GCTGGTCAGCCATAACCTTGG |
|  | *MMP2* | CCTTCACTTTCCTGGGCAACA | AAGGTCAATGTCAGGAGAGGC |
|  | *PLAT* | CCACTGCTTCCAGGAGAGGTT | AGGAGACAAGGCCTCATGCTT |
|  | *PLAU* | TCTGTCACCTACGTGTGTGGA | ATACATCGAGGGCAGGCAGAT |
|  | *ACE* | GATGGACACCACAGAGGCTAT | AAGGCCACAGGTAAGTCTTTG |
|  | *CTSB* | CTCTGCTGCCTGCTGGTGTT | CGGTCAGAGATGGCTTCCAC |
| qPCR | 18S rRNA | CGGACAGGATTGACAGATTG | CAAATCGCTCCACCAACTAA |
|  | *Gapdh* | GGCAAATTCAACGGCACAGT | AGATGGTGATGGGCTTCCC |
|  | *Il1b* | TTCAGGCAGGCAGTATCACTC | GAAGGTCCACGGGAAAGACAC |
|  | *Tnfa* | CCAGTGTGGGAAGCTGTCTT | AAGCAAAAGAGGAGGCAACA |
|  | *Il6* | TAGTCCTTCCTACCCCAATTTCC | TTGGTCCTTAGCCACTCCTTC |
|  | *iNOS* | GGAGTGACGGCAAACATGACT | TAGCCAGCGTACCGGATGA |
|  | *Cd200r1* | TAAGGTGGAGGCATTTCCAGT | GATTCCAATGGCCGACAAAGTA |
|  | *Sirpa* | CTCTCCGCGTCCTGTTTCTG | TCTGTACCACCTAATGGGTCC |
|  | *Tgfbr2* | GACTGTCCACTTGCGACAAC | GGCAAACCGTCTCCAGAGTAA |
|  | *Mme* | ACTGATTCGTCAGGAACAAAGTT | GGTCATTTCGGTCTTCTGGTT |
|  | *Ide* | AATCCGGCCATCCAGAGAATA | GGGTCTGACAGTGAACCTATGT |
